# Structural and thermodynamic characterization of a highly amyloidogenic dimer of transthyretin involved in a severe cardiomyopathy

**DOI:** 10.1016/j.jbc.2024.107495

**Published:** 2024-06-24

**Authors:** Lucas do Amaral Martins, Priscila S. Ferreira, Otávio Augusto Leitão dos Santos, Leticia Oliveira Martins, Luiz Gabriel Cabral Fernandes Barroso, Humberto M. Pereira, Márcia Waddington-Cruz, Fernando Lucas Palhano, Debora Foguel

**Affiliations:** 1 Instituto de Bioquímica Médica Leopoldo De Meis, Universidade Federal do Rio de Janeiro, Rio de Janeiro, Brazil; 2 Instituto de Física de São Carlos, Universidade São Paulo, São Carlos, Brazil; 3 Centro de Estudos de Paramiloidose Antônio Rodrigues de Mello, Hospital Universitário Clementino Fraga Filho, Universidade Federal do Rio de Janeiro, Rio de Janeiro, Brazil

**Keywords:** Transthyretin, amyloidosis, protein aggregation, protein structure

## Abstract

Transthyretin (TTR) is an homotetrameric protein involved in the transport of thyroxine. More than 150 different mutations have been described in the TTR gene, several of them associated with familial amyloid cardiomyopathy. Recently, our group described a new variant of TTR in Brazil, namely A39D-TTR, which causes a severe cardiac condition. Position 39 is in the AB loop, a region of the protein that is located within the thyroxine-binding channels and is involved in tetramer formation. In the present study, we solved the structure and characterize the thermodynamic stability of this new variant of TTR using urea and high hydrostatic pressure. Interestingly, during the process of purification, A39D-TTR turned out to be a dimer and not a tetramer, a variation that might be explained by the close contact of the four aspartic acids at position 39, where they face each other inside the thyroxine channel. In the presence of subdenaturing concentrations of urea, bis-ANS binding and dynamic light scattering revealed A39D-TTR in the form of a molten-globule dimer. Co-expression of A39D and WT isoforms in the same bacterial cell did not produce heterodimers or heterotetramers, suggesting that somehow a negative charge at the AB loop precludes tetramer formation. A39D-TTR proved to be highly amyloidogenic, even at mildly acidic pH values where WT-TTR does not aggregate. Interestingly, despite being a dimer, aggregation of A39D-TTR was inhibited by diclofenac, which binds to the thyroxine channel in the tetramer, suggesting the existence of other pockets in A39D-TTR able to accommodate this molecule.

Transthyretin (TTR) is an homotetrameric β-sheet–rich protein mostly found in the plasma (0.2 mg/ml; 3.63 μM) but also in the cerebrospinal fluid (1, 2), where it is the main thyroxine transporter of the brain. While the TTR localized in the brain is produced in the choroid plexus, plasma TTR is mainly produced by the liver and, besides its participation in thyroxin transport, it also binds to the retinol-binding protein forming a ternary complex with retinol (2).

TTR consists of four identical subunits of 127 amino-acid residues each, arranged in eight antiparallel β strands lettered A to H, in addition to a short α-helix between β sheets E and F (2). The monomers organize themselves into two dimers (AB and CD) through hydrogen bonds between H and F strands (3). Two dimers interact to form a tetrameric structure by the contacts between the β sheets DAGH (4). Two thyroxine-binding channels are formed in the dimer–dimer interface to accommodate thyroxine, each one with three symmetrical halogen-binding pockets into which the four iodine atoms of the ligand are lodged (5). Small ligands have been designed to fit into the thyroxine channels, some of them with anti-amyloidogenic properties due to their tetramer-stabilizing effect (6, 7, 8, 9, 10, 11, 12, 13).

More than 150 variants of TTR have been described worldwide and most of them are involved in three TTR-related amyloid diseases (ATTR), namely, familial amyloid cardiomyopathy, familial amyloid polyneuropathy, and a rare form of amyloidosis called oculoleptomeningeal amyloidosis (14, 15, 16). WT-TTR is also involved in an age-related form of ATTR known as senile systemic amyloidosis (ATTR-WT) in which amyloid deposits are mainly found in the heart (17).

Among the variants, V50M-TTR (formerly V30M; in the present study, we have considered the signal peptide 20 amino acids long as part of the primary sequence of TTR (18)) is the most prevalent worldwide. It is found in Portugal, where it is endemic, in Japan, Sweden (19, 20) and in Brazil (21), with the patients presenting severe neuropathic manifestations. V142I-TTR patients (formerly V122I), which affects 3 to 4% of the African-American population, present severe cardiac complications (22). In addition to these widely known tetrameric variants of TTR, there are others, such as D38G (formerly D18G) and S132I-TTR (formerly S112I), the only ones described so far that involve nontetrameric TTR: D38G-TTR is a monomer while S132I-TTR is a dimer. As expected, they are both highly amyloidogenic and associated with pathologies (23, 24). The dimers of S132I aggregate more readily and in a broader pH range when compared to the WT-TTR. However, aggregation of the dimer is not inhibited by TTR stabilizers such as thyroxine (its natural ligand) and flufenamic acid (a nonsteroidal anti-inflammatory compound) since S132I-TTR, as a dimer, does not have the thyroxine-binding pockets (24).

Nontetrameric structures of TTR are rare (only these two described so far) and dissection of the dissociation-aggregation pathways of these nontetrameric TTRs is relevant to the full understanding of how TTR aggregation takes place. The accepted mechanism for TTR aggregation into amyloid fibrils postulates first tetramer dissociation into monomers, the rate-limiting step of the process, and the further partial unfolding of the separated monomers, which then form oligomers and mature amyloid fibrils (25, 26, 27, 28, 29, 30). Thus, nontetrameric variants of TTR, which are essentially en route to fibril formation, tend to have a great propensity for amyloidogenesis.

In the last few years, our group has been studying extensively the folding and aggregation processes of TTR *in vitro* and *in silico*. In our University Hospital, we have a referral center for the diagnosis of ATTR and a facility for conducting follow-up studies with the patients (31, 32, 33, 34, 35, 36, 37, 38, 39). Recently, we described a novel mutation in the TTR gene (A19D-TTR, now A39D-TTR) in a Brazilian male with German ancestry living in southern Brazil who died from a severe cardiomyopathy (37). In this first report, the stability of this new variant was studied *in silico* by using bioinformatics tools (FoldX force field) and compared to those of T139M-TTR (formerly T119M), a very stable and nonamyloidogenic variant of TTR, and V50M-TTR. The predicted decrease in thermodynamic stability of the supposed tetramers of A39D-TTR in relation to WT-TTR was calculated to be approximately 11 kcal/mol. The stability loss was mostly associated with the dissociation of tetramers into dimers and not with the dissociation of dimers into monomers. Since position 39 is in the AB-loop, which is part of the thyroxine-binding channel, we envisioned that the four negative charges displayed by the aspartic acid residues facing each other inside the channel would cause electrostatic repulsion affecting tetramer stability.

In the present study, A39D-TTR was cloned and purified for *in vitro* studies. Its thermodynamic stability and aggregation propensity were probed and compared to that of WT-TTR. Surprisingly, in solution, this variant of TTR exists as a folded dimer, which has an increased propensity to aggregate into amyloid fibrils in a broader pH range (pH 4–5.8) and with a faster rate than WT-TTR. Aggregation of A39D-TTR is inhibited by diclofenac, suggesting that the anti-amyloidogenic compounds that can inhibit the aggregation of TTR through tetramer stabilization might find other binding site(s) besides the thyroxine-binding channels that contribute to TTR aggregation. The crystal structure of A39D-TTR revealed that the negative charges of the four aspartic acids would be in very close proximity inside the thyroxine channel of a hypothetical tetramer of A39D-TTR, thus indicating that charge repulsion in the dimer–dimer interface might hinder the formation of tetramers of this variant. Denaturation by urea and by high hydrostatic pressure (HHP) confirmed the decreased stability of the dimer, and the thermodynamic parameters (ΔGº and ΔVº) associated with dimer-monomer equilibrium were assessed. Bis-ANS binding experiments in the presence of low concentrations of urea revealed the existence of a partially denatured dimer, a structure not previously reported in TTR denaturation pathway. Complete characterization of the dissociation pathway of this dimer into monomers allowed us to explore this transient step in the TTR dissociation pathway (tetramer→[dimer]→monomer), bringing new insights to the understanding of TTR dissociation and amyloid formation.

## Results

### A39D-TTR is a dimer in solution

In our previous work with A39D-TTR (37), bioinformatics analysis (FoldX force field) predicted a large decrease in the stability of this variant of TTR, when compared to the WT-TTR, by 10.9 kcal/mol. For that analysis, we assumed that A39D-TTR was a tetramer and, according to FoldX, this decrease in stability was more related to the dissociation of the tetramers into dimers than to the dissociation of dimers into monomers. Thus, we inferred that the four negative charges from the aspartic acids that face the dimer–dimer interface (AB/CD) might cause electrostatic repulsion, affecting tetramer stability.

**Figure 1:**
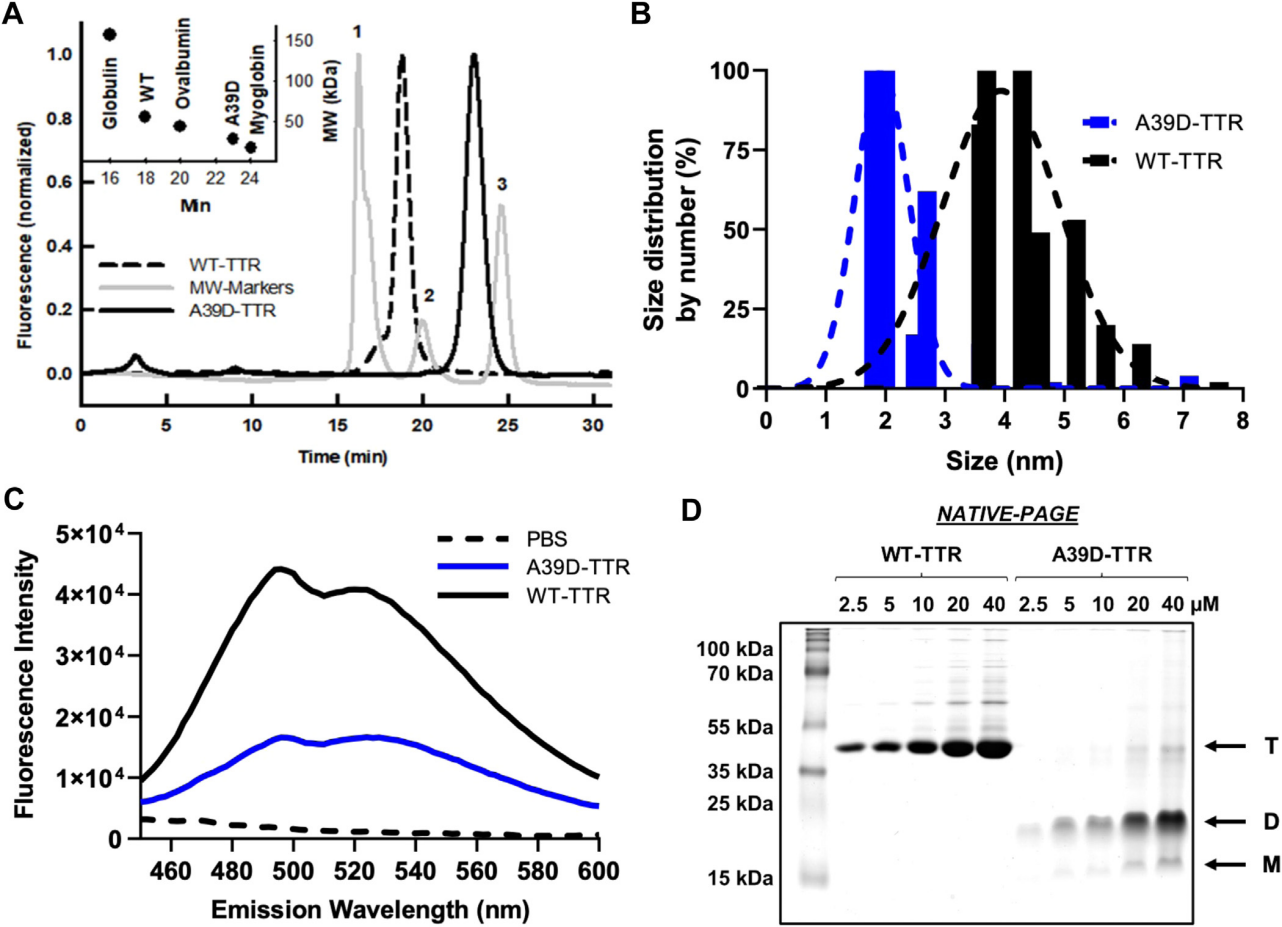
**A39D-TTR is a dimer and not a tetramer in solution.***A*, SEC elution profile in 25 mM Tris HCl, 100 mM KCl, and 1 mM EDTA (pH 8.0) of WT-TTR (*black dashed line*), A39D-TTR (*black line*), and molecular weight markers (*gray line*): 1. δ-globulin (158 kDa); 2. ovalbumin (44 kDa); and 3. myoglobin (17 kDa). *B*, DLS size distribution of 10 μM of A39D-TTR (*blue bars* and *line*) and 10 μM WT-TTR (*black bars* and *line*). *C*, fluorescence emission spectra of 10 μM VBO in the presence of 4 μM A39D-TTR (*blue line*) and 2 μM WT-TTR (*black line*). *Dashed* line shows VBO in PBS. *D*, Native-PAGE of WT (2.5–40 μM) and A39D-TTR (2.5–40 μM).

To investigate this in depth, A39D-TTR was cloned and purified to homogeneity for structural, thermodynamics, and aggregation studies. During the process of purification, we noticed its anomalous behavior in size-exclusion chromatography (SEC) (Fig. 1*A*). Whereas at pH 8.0, the WT-TTR eluted at ∼18 min (dashed line), which corresponds to a protein of ∼56 kDa (inset, panel A), A39D-TTR eluted at 23 min (continuous black line), an elution time between ovalbumin (44 kDa; 20 min; peak #2) and myoglobin (17 kDa; 24 min; peak #3), which is compatible with the mass of TTR dimer (28 kDa, inset, panel A), suggesting that A39D-TTR is not a tetramer like the other TTR variants.

To verify the proposition that A39D-TTR is indeed a dimer, dynamic light scattering (DLS) measurements were performed (Fig. 1*B*). DLS reinforced the idea of a dimeric A39D-TTR, with a mean radius of 1.9 nm (blue distribution curve and blue bars), compatible with a protein of 28 kDa, while the tetramer of WT-TTR displayed a radius of 3.9 nm (black distribution curve and black bars) compatible with a molecular weight of 56 kDa. The correlation curves of A39D-TTR and WT-TTR (polydispersity = 0.390 and 0.432, respectively) are displayed in Fig. S1.

To confirm that this new mutation is in fact a dimer and does not form a tetramer in the presence of molecules that bind into the thyroxine-binding channel, we analyzed the fluorescence of VBO, a probe that binds specifically to the thyroxine-binding channel. This molecule (2-[(3,5-chlorophenyl)amino]benzoic acid) is a structural analog of diclofenac (a non-steroidal anti-inflammatory), which fluoresces when bound to the thyroxine-binding channel (11, 40), indicating the presence of TTR tetrameric structures. As seen, the emission of VBO (10 μM) in the presence of A39D-TTR (Fig. 1*C*, blue line) presented a minor increase, unlike the pronounced increase when this probe binds to thyroxine channels in the WT-TTR (Fig. 1*C*, black line) reinforcing the dimeric nature of A39D-TTR. However, this slight increase in the fluorescence emission of VBO in the presence of A39D-TTR suggests that there might be noncanonical-binding site(s) on the variant for this probe or even that a small population of A39D-TTR is being converted into tetramers.

As expected, when both proteins were subjected to SDS-PAGE, they showed a major band at similar weights (∼15 kDa) related to TTR monomers (Fig. S2*M*). Furthermore, Fig. S2 also shows that while WT-TTR also presented faint bands with molecular weights compatible with the dimer (∼28 kDa; D) and tetramer (∼56 kDa; T), A39D-TTR presented another faint band compatible with the dimer and an almost imperceptible band related to the tetramers. In a Native-PAGE, WT-TTR presented a major band related to the tetramer; however, A39D-TTR did not show this band, but a major band at the bottom of the gel compatible with a lower molecular weight species (Fig. 1*D*). Increasing the concentration of A39D-TTR to 20 and 40 μM, we start to see a faint band in a position compatible with the tetramer, but this band represents less than 5% of the total protein species present. To investigate whether A39D-TTR could be forming a tetramer at higher protein concentrations, we measured the binding of VBO to A39D-TTR varying protein concentration from 2.5 to 40 μM with either equimolar or double VBO concentrations. As seen in Fig. S3, there was no binding of VBO to A39D-TTR at any protein or VBO concentrations.

Taken together, these results strongly corroborate the proposition that A39D-TTR is a dimer of TTR, which would explain its decreased thermodynamic stability, as predicted by FoldX. The four negative charges that face each other within the thyroxine-binding channel might impede the tetramerization of the protein, explaining its dimeric nature.

### The crystal structure of A39D-TTR explains its dimeric nature

We solved the crystal structure of A39D-TTR to search for the structural alterations caused by the mutation, which lies within the thyroxine-binding channel. Our previous *in silico* molecular model of A39D-TTR superimposed on the WT-TTR structure (PDB 1F41) suggested the proximity of the four negative charges of the D39 side chains inside the channel (37).

A39D-TTR crystallizes in space group P21212 with one TTR dimer in the asymmetric unit. A summary of data collection, processing, and refinement is presented in the structure deposited in the PDB bank as entry 5DEJ.

**Figure 2:**
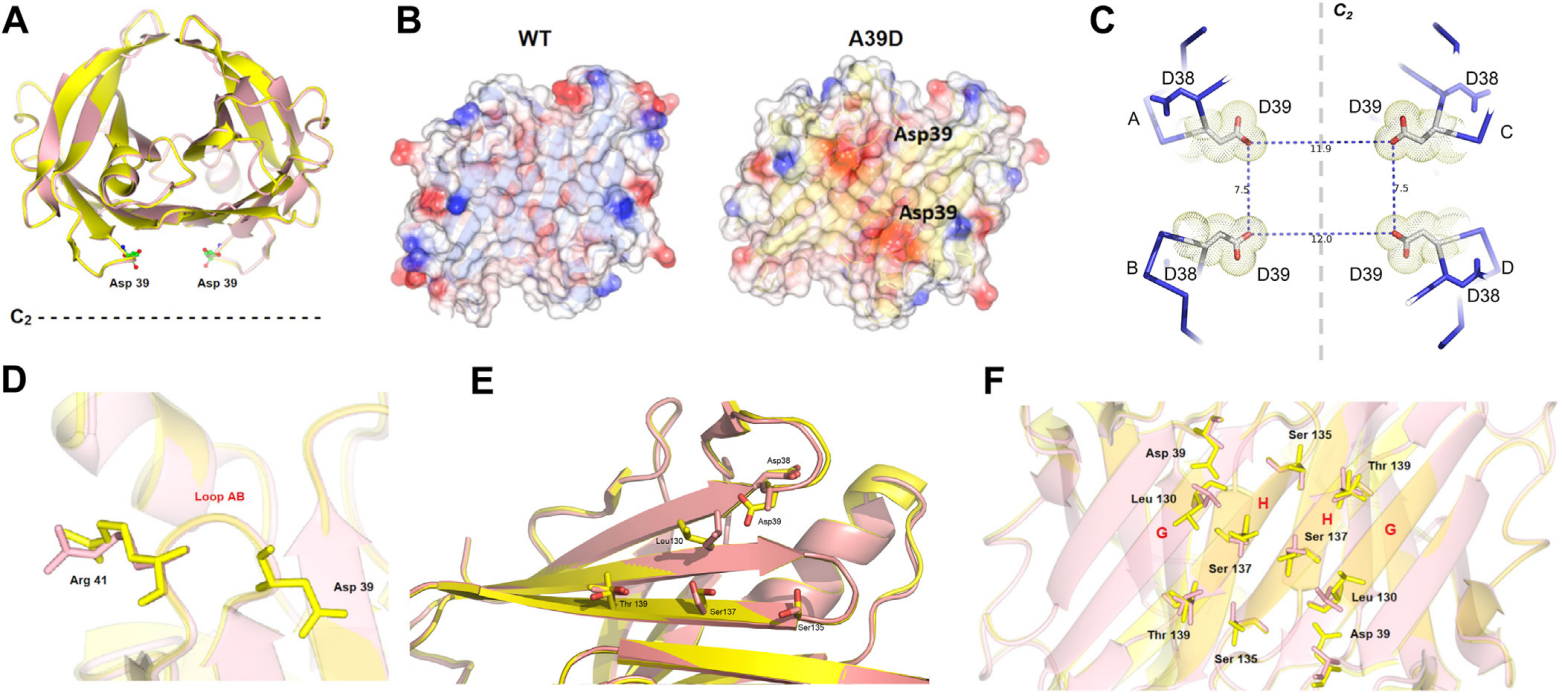
**Crystallographic structure of A39D-TTR at pH 7.0.***A*, the AB subunits of A39D-TTR (PDB 5DEJ, *yellow*) are superimposed onto the structure of WT (PDB 1F41, *magenta*). *B*, electrostatic map of AB subunits of A39D-TTR and WT where it is possible to see the two negative charges in *red* of the two aspartic acids located at position 39. In *blue*, positively charged amino acid. *C*, A39D-TTR structure showing the distances in Å between the four Asp 39 in the C_2_ axis. *D*, close look of the AB loop showing the local modification of Arg41 that moves towards Asp 39 in A39D-TTR. *E*, close look at strands H and G in the A39D-TTR (*yellow*) showing the changes in the interactions among the residues Leu130, Ser135, Ser137, and Thr 139 in relation to the WT-TTR structure (*magenta*). *F*, details of dimeric interface in C_2_ axis of the structures A39D-TTR and WT-TTR.

Figure 2*A* shows the overall structure of the AB subunits of A39D-TTR (yellow) superimposed onto the structure of the WT-TTR (PDB 1F41, magenta), where it is possible to see the similarity between the structures (r.m.s.d. of 0.486 Å). The two aspartic acid residues from the A and B subunits face the C2 axis, which crosses the thyroxine channels.

Figure 2*B* shows the electron density maps of the WT-TTR and A39D-TTR, where the mutation is readily identified in the latter due to its negative character, which is absent in the WT-TTR structure. Position 39 is in the AB-loop (residues 37–43), a loop that faces the thyroxine-binding channels being involved in TTR tetramerization (dimer-dimer interface; AB/CD). It is expected that in a tetramer, the presence of four negative charges in proximity inside these channels might cause electrostatic repulsion, perturbing protein–protein interaction. Figure 2*C* shows images of the thyroxine channels highlighting the four aspartic acids and the distances between the acidic groups. It should be noted that position 38 in TTR is occupied by an aspartic acid (D38), which might exert a repulsive force against D39. However, D38 points in another direction in the structure (Fig. 2*E*). The side chain of D38 interacts with V40, R41, and Y98 (not shown), contributing to the structure of the loop 38 to 43.

The superposition of the A39D-TTR structure (yellow) on WT-TTR (magenta) shows that in the AB-loop, R41 is displaced toward D39 (Fig. 2*D*), probably due to charge attraction. Indeed, a close look at the region surrounding the mutation shows some side-chain rearrangements. The side chain of L130, which in WT-TTR structure points towards A39, undergoes a steric clash with D39 forcing L130 to assume a different rotamer (Fig. 2*E*). Figure 2*F* shows details of β-strands H and G, which lie in the dimeric interface of the crystallographic C2 axis, highlighting the repositioning of the residues of A39D-TTR due to the mutation. Residues S135, S137, and T139, which lie in the β-strand H, also show different conformations in comparison to their position in the 1F41 structure (Fig. 2*F*). Altogether, these structural modifications might interfere with tetramerization of the A39D-TTR.

### Probing the thermodynamic stability of A39D-TTR dimers

**Figure 3:**
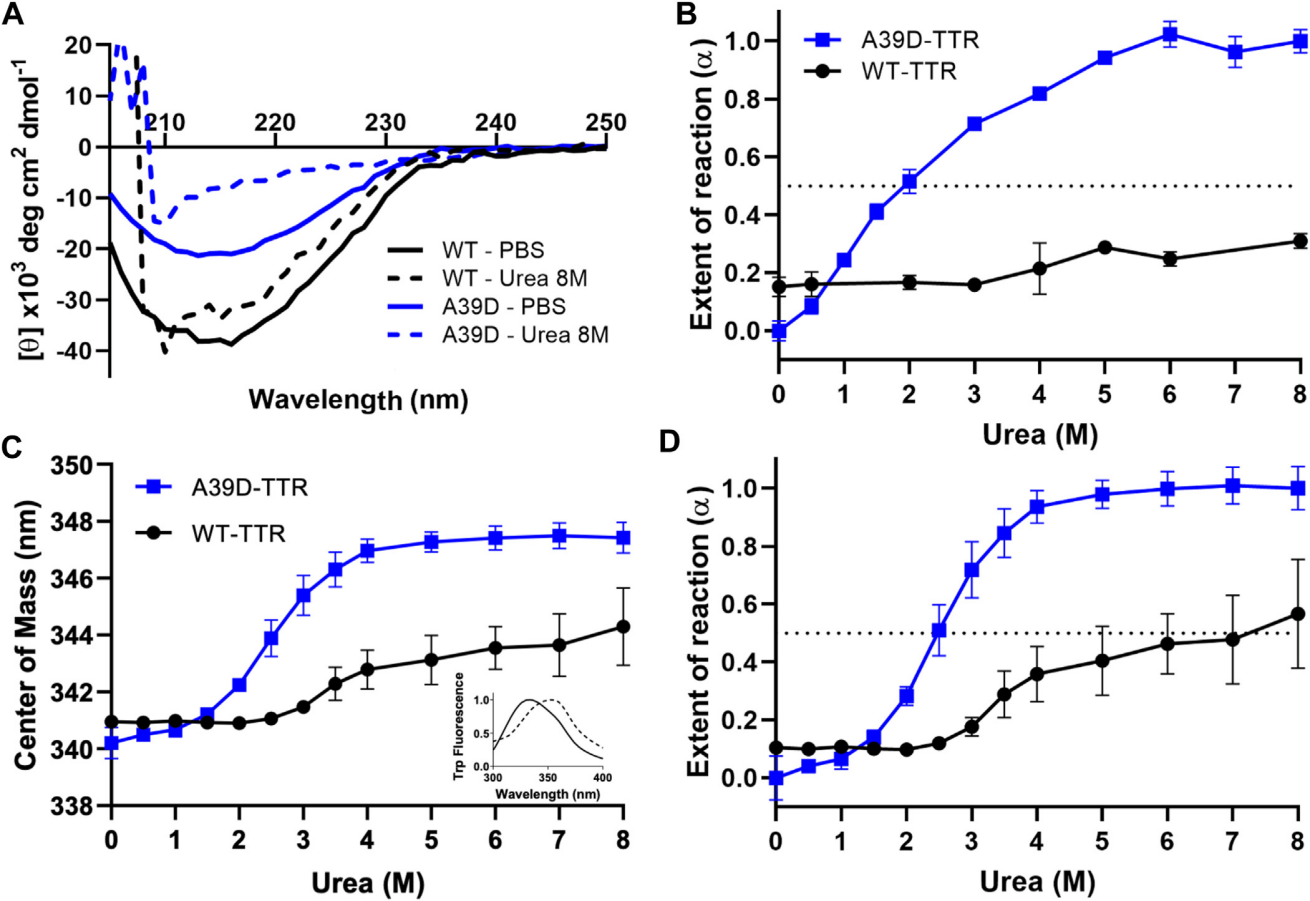
**Urea-induced unfolding of WT- and A39D-TTR as revealed by circular dichroism and tryptophan fluorescence emission.***A*, spectra of WT- (*black lines*) and A39D-TTR (*blue lines*) in the absence (*solid lines*) and in the presence of 8 M urea (*dashed lines*). *B*, the extent of unfolding (α) as a function of the urea concentration, extracted from the circular dichroism data. *C*, changes in the center of mass of tryptophan fluorescence as a function of urea concentration. The inset shows the fluorescence emission spectra of A39D-TTR in the absence (*solid line*) and in the presence of 8 M urea (*dashed line*). *D*, the extent of unfolding (α) as a function of urea concentration extracted from the tryptophan fluorescence emission spectra. The protein concentrations used in all experiments were 4 μM for A39D-TTR and 2 μM for WT-TTR. The experiments were performed at pH 7.4 at 25 °C after 96 h in the presence of urea and repeated at least three times.

**Figure 4:**
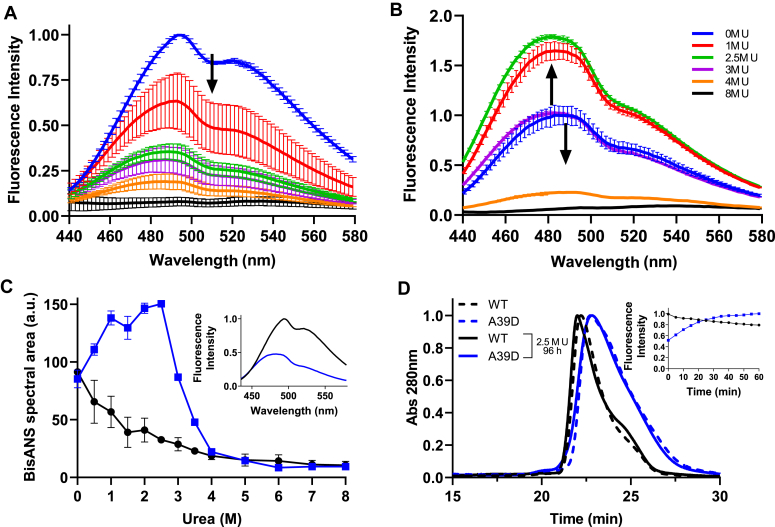
**Changes in the tertiary structure of WT- and A39D-TTR induced by different concentrations of urea as monitored by bis-ANS binding.***A* (WT-TTR) and *B* (A39D-TTR) show bis-ANS emission spectra as a function of urea concentration (96 h incubation at pH 7.4 and 25 °C). Line colors represent: 0 M urea (*blue*), 1 M urea (*red*), 2.5 M urea (*green*), 3 M urea (*purple*), 4 M urea (*yellow*), and 8 M urea (*black*). Bis-ANS fluorescence emission was measured by exciting the samples at 385 nm and collecting the emission from 435 to 580 nm. *C*, bis-ANS spectral area for the data presented in panels *A* and *B*. A39D-TTR (*blue line*) and WT (*black line*). The inset shows bis-ANS spectra of WT-TTR (*black line*) and A39D-TTR (*blue line*) in the absence of urea ([bis-ANS] = 10 μM, [A39D-TTR] = 4 μM, and [WT-TTR] = 2 μM). *D*, SEC profiles in a SD-75 column equilibrated in 2.5 M urea showing the elution times of WT-TTR (10 μM; *black lines*) and A39D-TTR (20 μM; *blue lines*) in the native states (*dashed lines*) or after 96 h in the presence of 2.5 M urea (*solid lines*). Elution was monitored at 280 nm. The elution times are different from those presented in Figure 1*A* due to the presence of urea in the mobile phase. The inset shows the kinetics of bis-ANS binding to A39D-TTR (*blue*) and to WT-TTR (*black*) as revealed by fluorescence emission when the proteins were placed in the presence of 2.5 M urea. The experiments were repeated at least three times.

The unfolding of A39D-TTR induced by urea was investigated by following secondary (circular dichroism) and tertiary (tryptophan and bis-ANS emission) structures (Figs. 3 and 4). In panel A of Figure 3 is presented the circular dichroism spectra of the WT- (black lines) and A39D-TTR (blue lines) in the absence of urea (full lines) and after 96 h in the presence of 8 M urea (dashed lines). As seen, when native, both proteins presented the typical β-sheet–rich spectra. However, while 8 M urea denatured the variant almost completely, the secondary structure of the WT-TTR remained quite stable. In panel B, the complete urea denaturation curves are expressed as the extent of reaction (α; Equation 1, Experimental procedures) for both proteins at the same monomer concentration (2 μM A39D- and 1 μM WT-TTR), where it is possible to see that at 6 M urea, A39D-TTR is completely denatured, with a C_m_ (urea concentration that furnishes 50% denaturation) of 2 M. The WT secondary structure remained almost unaltered, even in high urea concentrations, if we assume that the maximum denaturation is the signal presented by the variant at 8 M urea (panel A, dashed blue line).

In panels C and D (Fig. 3) are presented, respectively, the shifts to the red in tryptophan maximum fluorescence emission (Equation 2, Experimental procedures), and the extent of reaction (α; Equation 1), as a function of urea addition. The concentrations of A39D- and WT-TTR were equals in terms of monomers (2 μM A39D- and 1 μM WT-TTR). Regarding the tertiary structure alterations, 4 M urea was enough to denature completely A39D-TTR (C_m_ value of 2.5 M), while WT-TTR was only partially denatured even at 8 M urea (∼50%, panel D). If we consider the final center of mass of tryptophan emission of WT-TTR as a final step of its urea-induced denaturation (344 nm, panel C), the C_m_ value was equal to 3.6 M (see Fig. 6*E* for clarity).

Next, we probed bis-ANS binding to WT- and A39D-TTR as a function of urea-induced denaturation (Fig. 4). Bis-ANS has been used to map partially denatured or molten-globule states of proteins. When bound to hydrophobic pockets in proteins, this probe displays a great increase in its fluorescence emission (41, 42). As previously shown, bis-ANS can bind into the thyroxine-binding channels of TTR (31, 43) and probably in other hydrophobic cavities in the structure of the protein.

In the case of the WT-TTR, there was a progressive decrease in bis-ANS emission when the concentration of urea was increased, suggesting perturbation in the thyroxine-binding channels, probably related to tetramer dissociation, and partial unfolding of the separated monomers (panels A and black circles in C). Interestingly, in the native state, the dimer of A39D-TTR (inset of panel C, blue line) also binds bis-ANS, although with less fluorescence emission, when compared to the WT protein (inset of panel C, black line) at an identical bis-ANS:monomer ratio. Considering the absence of thyroxine channels in the dimeric structure, bis-ANS binding to A39D-TTR might be associated with other pockets able to accommodate this probe in the native state of the TTR dimer. Interestingly, the addition of urea up to 2.5 M led to an increase in bis-ANS binding (panel B), suggesting the formation of a molten-globule–like conformation able to accommodate even higher concentrations of this probe. From 3 to 4 M urea, there was a progressive decline in bis-ANS emission suggesting complete denaturation of the dimers (panels B and blue squares in C).

To map in more detail which species (monomers or partially denatured dimers) of A39D-TTR would be formed in the presence of subdenaturing concentrations of urea (up to 3 M urea), SEC was performed with the column equilibrated with 2.5 M urea. As seen in Figure 4*D*, A39D-TTR incubated in the presence of 2.5 M urea for 96 h eluted at the same time as the native A39D-TTR dimer (blue curves), suggesting that the quaternary structure of the proteins seems to be preserved in this urea concentration. The elution profile of WT-TTR incubated in the presence of 2.5 M urea for 96 h is also presented, where only the tetramer is seen (black curves). DLS measurements with A39D-TTR incubated in the presence of 2.5 M urea for 96 h, a concentration of urea which already induces large amounts of bis-ANS binding, revealed the presence of a species with 4.0 to 4.5 nm mean size (data not shown), a dimension compatible with an expanded dimer but not with a monomer (compare with the data presented in Fig. 1*B*). Thus, low concentrations of urea seem to induce the formation of a molten-globule dimer, which binds more bis-ANS than the native one, having partially lost its secondary and tertiary structures. Other structural observations are necessary to ensure that A39D-TTR is indeed in a molten-globule conformation in subdenaturing concentrations of urea.

To determine how fast is the transition from a native dimer to this molten globule dimer, the kinetics of bis-ANS binding in the presence of 2.5 M urea was examined (inset of Fig. 4*D*, blue line). Thus, A39D-TTR was incubated in 2.5 M urea in the presence of bis-ANS and fluorescence intensity at 485 nm was monitored over time to follow bis-ANS binding. As seen, in ∼30 min, in the presence of 2.5 M urea, the bis-ANS–binding capacity of A39D-TTR almost doubled, reflecting a fast transition to the altered dimeric conformation, while the bis-ANS–binding capacity of WT-TTR (inset of Fig. 4*D*, black line) slightly decays, indicating the dissociation of the tetramers and loss of bis-ANS binding as seen in Figure 4*A*. Thus, it is possible that the native dimer, when injected into an SEC column equilibrated with 2.5 M urea, converts into this molten-globule dimer during the ∼30 min of elution.

**Figure 5:**
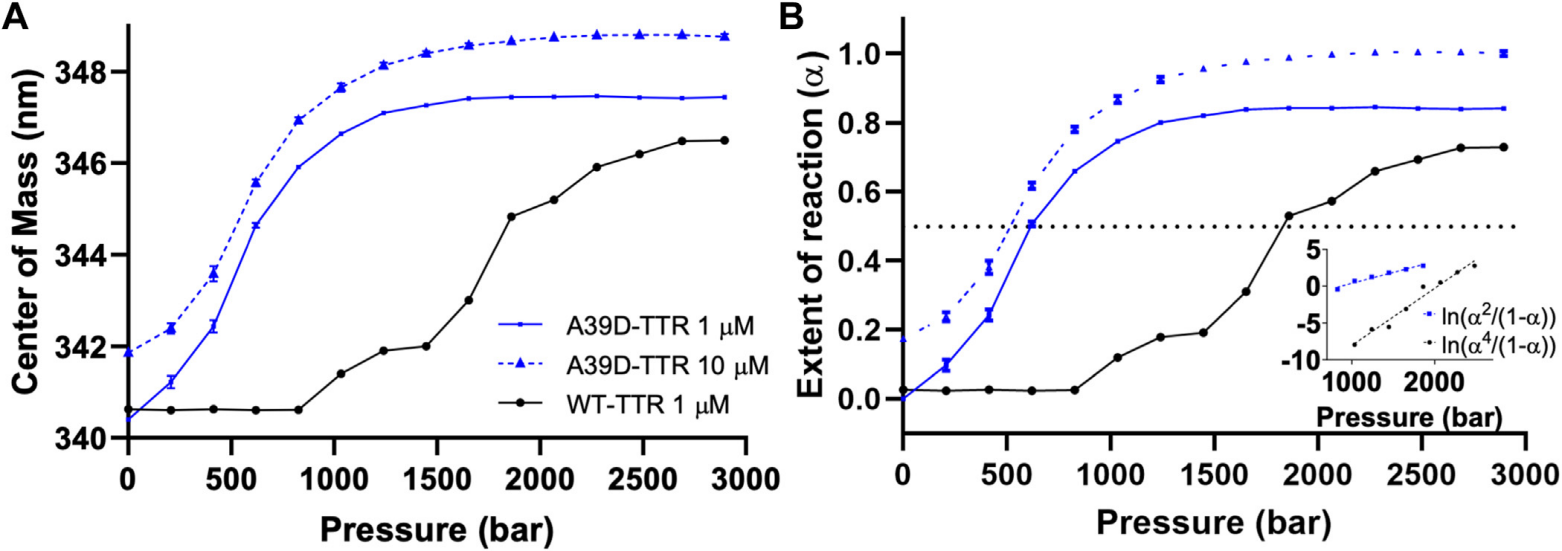
**High hydrostatic pressure–induced dissociation-unfolding of WT- and A39D-TTR as revealed by tryptophan fluorescence emission.***A*, center of mass change of tryptophan emission as a function of pressure for A39D-TTR (*blue lines*) and WT-TTR (*black line*). *B*, the extent of reaction (α) as a function of pressure for the data presented in panel *A*. The protein concentrations were 1 and 10 μM for A39D-TTR (*blue squares* and *triangles*, respectively) and 1 μM WT-TTR (*black circles*). The inset of panel (*B*) presents the linear regression for the data presented in panel B as described in Equations 3 and 4. The data for A39D-TTR are in *blue* (ln (α^2^)/(1- α)) and for WT-TTR in *black* (ln (α^4^)/(1-α)). The experiments were carried out at pH 7.0 at 1 °C and repeated at least three times (the error bars have the same size of the symbols). Tryptophan emission was measured with excitation at 280 nm and emission collected from 300 to 400 nm.

Next, we probed the stability of A39D-TTR by HHP, which has been used extensively to dissociate/unfold proteins, including TTR, allowing the calculation of the thermodynamic parameters such as volume and free-energy changes of association (ΔVº and ΔGº) (24, 31, 32, 34, 40, 44). Figure 5*A* shows HHP dissociation curves for WT-TTR (1 μM) and A39D-TTR (1 μM and 10 μM) at pH 7.0, 1 °C. As shown before, low temperatures facilitate the dissociation of TTR, a very stable tetramer (31, 45, 46). The center of spectral mass of tryptophan emission was used as a sensor of the structural changes induced by HHP, and the data are expressed as the extent of reaction (α) calculated according to Equation 1 (Fig. 5*B*). As seen in Figure 5*A*, at 2.4 kbar, there was a red shift to approximately 346 to 348 nm in the emission of WT- and A39D-TTR, suggesting pronounced exposure of the tryptophan to the aqueous environment (panel A). In the case of HHP-induced dissociation/denaturation, both WT and the variant achieved almost the same extent of tryptophan exposure, which was not observed in the presence of high concentrations of urea. The p_1/2_ values (pressure that furnishes 50% changes in tryptophan emission) were equal to 600 bar and 1700 bar for A39D-TTR (blue squares) and WT-TTR (black circles), respectively, confirming the lower stability of the variant (panel B). After decompression, the initial center of spectral mass values of tryptophan emission recovered completely (data not shown), suggesting complete reversibility of the dissociation/denaturation processes in all cases.

The dissociation of dimers is in general concentration-dependent although some exceptions have been described in the literature (41, 47). As shown in Figure 5, the dissociation of A39D-TTR did not present any dependence on concentration, which has also been observed to some extent with the dissociation of tetramers of WT-TTR.

To access the thermodynamic parameters for the association reactions of WT- and A39D-TTR, the plots ln ((α^4^)/(1-α)) and ln ((α^2^)/(1-α)) *versus* pressure, respectively, were constructed and are presented in the inset of panel B. From these plots, the ΔVº (slope of the curve) and ΔGº of association (intercept on the y-axis) were extracted (Equations 3 and 4). Regarding the ΔVº of association, the values found were 177.12 ml/mol (WT-TTR) and 67.25 ml/mol (A39D-TTR), while the ΔGº of association values were, respectively, −28.14 kcal/mol and −7.78 kcal/mol at 1 °C.

### Is it possible to form hetero-tetramers or hetero-dimers composed of WT and A39D subunits to mimic the patient’s condition?

Patients of FAC that present TTR mutations usually are heterozygous, and thus, most of their tetramers and/or dimers are, in principle, hetero-tetramers (h-T) or hetero-dimers (h-D) assuming they are equally produced and secreted by the liver/choroid plexus cells, if the mutation does not compromise severely the stability of the protein during its folding in the endoplasmic reticulum (48).

**Figure 6:**
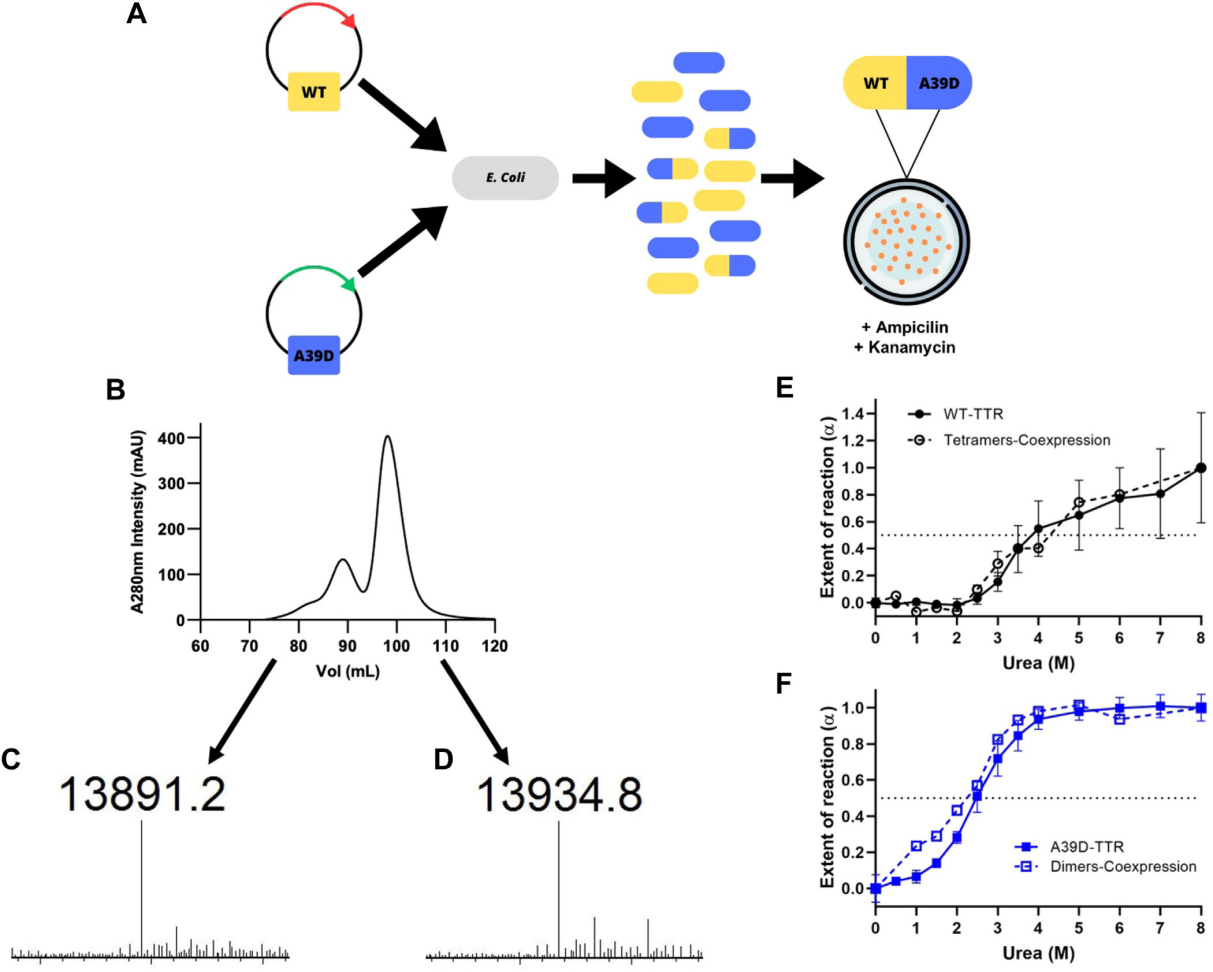
**Cotransformation assays with WT- and A39D-TTR plasmids does not reveal the formation of hetero-tetramers (h-T) or hetero-dimers (h-D).***A*, schematic representation of the co-transformation protocol used. Plasmids of WT-TTR (*yellow*; resistant to Ampicillin) and A39D-TTR (*blue*; resistant to Kanamycin) were inserted in the same bacterial cell (*E. coli* BL21 DE3). Bacteria were grown in the presence of both antibiotics to select those cells that incorporated the two plasmids (*yellow*/*blue*). *B*, SEC profile of the TTR purification from the cotransformed bacterial cells, where two peaks corresponding to the tetramers and dimers are present. Each peak was carefully collected and subjected to mass spectrometry and their masses are displayed in panels (*C*) (tetramer) and (*D*) (dimer). Note that only the expected mass for the WT monomer was found in the peak related to the tetramers, while the expected mass for A39D-TTR monomer was found associated with the dimer peak. *E* and *F*, The WT- and A39D-TTR were purified from the cotransformed bacteria cells, purified to homogeneity, and incubated in the presence of increasing concentrations of urea as described previously (Fig. 3). Tryptophan fluorescence was recorded and converted into extent of reaction (α) and compared to that displayed by the WT-TTR (*E*) and A39D-TTR (*F*) expressed alone. The protein concentrations used were 4 μM A39D-TTR and 2 μM WT-TTR in all cases. Cotransfection was repeated at least three times with similar results.

To investigate whether h-T and h-D would be formed in a close physiological condition, we expressed the plasmids of A39D-TTR and WT-TTR in the same bacterial cell. Since these two plasmids exhibit different antibiotic resistances, the selection of bacterial cells that survive in the presence of both antibiotics allowed us to isolate those cells that incorporate both plasmids. These double-resistant bacterial cells were grown, protein expression was induced, and TTR was purified using the usual protocol for TTR purification (see the scheme in Fig. 6*A*). As seen in panel B, two main peaks emerged from the SEC column, the last step in TTR purification. Each peak was carefully collected, and the masses of the proteins were measured by mass spectrometry. The peak related to the elution of the tetramer (panel C) presented only one mass (13,891 Da), which is the mass expected from the WT monomer (13,890 Da). The mass of the peak eluting as a dimer (panel D) was precisely that of the A39D-TTR monomer (13,935 Da), with no other peak with the mass of the WT monomer. These data suggest that only homo-dimers or homo-tetramers are formed inside a bacterial cell co-expressing the two monomeric subunits of A39D-TTR and WT-TTR.

To confirm that the dimers and tetramers purified from the co-expression of WT- and A39D-TTR present the same thermodynamic stability as the A39D-TTR dimers and WT tetramers expressed separately, urea denaturation curves were performed with the purified co-expressed proteins, and they are presented in panels Figure 6, *E* and *F*. As seen, the dimers and tetramers purified after a co-expressing regime presented the same urea-denaturation profiles as their counterparts.

Taken together, these data suggest that the monomers of A39D-TTR cannot be incorporated into tetramers; when expressed, they are segregated as dimers. Also, tetramers do not seem capable of accommodating subunits of A39D-TTR. Further investigation would be necessary to understand this unexpected behavior.

### Probing amyloidogenicity of A39D-TTR dimers

**Figure 7:**
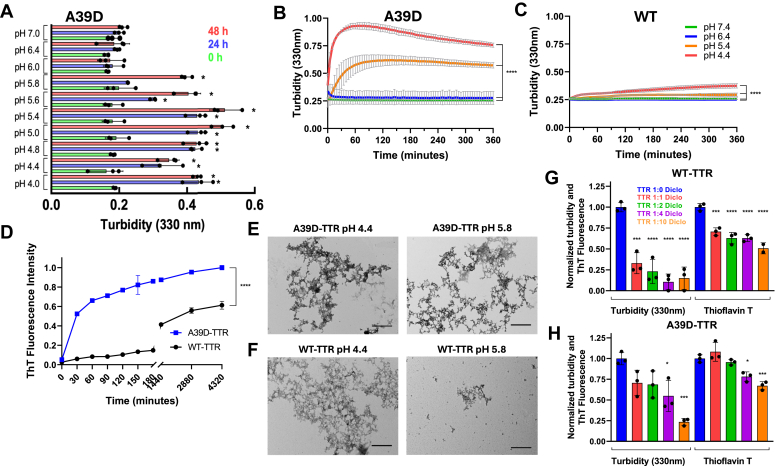
**A39D-TTR is highly amyloidogenic and its aggregation is inhibited by diclofenac.***A*, aggregation of A39D-TTR (10 μM) was measured by the turbidity at 330 nm at different pH values at different times (24 h; *blue* bars and 48 h; *red* bars). *B* and *C*, comparison of the aggregation profiles of A39D-TTR (10 μM) and WT-TTR (5 μM), respectively, at pH 7.4 (*green* line), pH 6.4 (*blue* line), pH 5.4 (*yellow* line), and pH 4.4 (*red* line). *D*, aggregation profiles of WT-TTR (*black*) and A39D-TTR (*blue*) revealed by thioflavin T (ThT) binding during 72 h. *E* and *F*, TEM images of A39D-TTR and WT-TTR, respectively, aggregated at pH 4.4 and 5.8 for 72 h as displayed. Calibration bar represents 500 nm. *G* and *H*, aggregation kinetics at pH 4.4 were performed with WT- (*G*; 5 μM) and A39D-TTR (H, 10 μM) for 72 h in the absence (*blue bars*) or in the presence of increasing concentrations of diclofenac (5 μM diclofenac, *red* line; 10 μM diclofenac, *green line*; 20 μM diclofenac, *purple line*; and 50 μM diclofenac, *orange line*). Turbidity at 330 nm and ThT binding were normalized considering the values obtained in the absence of any compound as 1. Data with a *p* value < 0.05 were considered significant. Asterisks denote *p* value, where ∗ = *p* < 0.05; ∗∗ = *p* < 0.01; ∗∗∗ = *p* < 0.001 ∗∗∗∗ = *p* < 0.0001.

Aggregation of WT-TTR is known to be very high at pH 4.4, requiring days for its completion (96 h). Next, we probed the aggregation profile of A39D-TTR by following the increase in turbidity at 330 nm in a broad pH range (pH 4–7) (Fig. 7*A*). As seen, A39D-TTR aggregated considerably from pH 4 to pH 5.8, even in shorter times such as 24 to 48 h. Panels B and C present the aggregation kinetics of A39D-TTR and WT-TTR, respectively, in short incubation times for comparison, where it is possible to see how fast A39D-TTR aggregates. In panel D is shown thioflavin-T (ThT; an amyloid specific probe) binding at pH 4.4 for A39D-TTR (blue squares) and WT-TTR (black circles), where it is possible to see that A39D-TTR forms ThT-positive aggregates in less than 3 h, an interval where WT-TTR did not aggregate at all. The TEM images confirm the formation of amyloid aggregates at pH 4.4 and 5.8, for both A39D- (Fig. 7*E*) and WT-TTR (Fig. 7*F*) aggregated during 96 h.

Finally, we probed whether diclofenac, a well-known TTR tetramer stabilizer, and an anti-amyloidogenic compound, due to its ability to bind to the thyroxine-binding channels, would have an impact on A39D-TTR aggregation. First, we seek to evaluate whether an A39D-TTR dimer would form a tetramer in the presence of diclofenac using an SEC equilibrated in buffer with 20 μM diclofenac. A39D-TTR (10 μM) and WT-TTR (5 μM) were previously incubated with diclofenac (20 μM) for 2 h at room temperature and the elution profiles are shown in Fig. S4, *A* and *B*, where it is possible to see that diclofenac did not change the dimeric state of A39D-TTR.

Interestingly, diclofenac inhibited the aggregation of A39D-TTR at pH 4.4 (96 h) in a dose-dependent manner as followed by turbidity at 330 nm and ThT fluorescence for both WT (panel G) and A39D-TTR (panel H). To assess the binding affinity of diclofenac for A39D-TTR, isothermal titration calorimetry (ITC) was performed (Fig. S5). The calculated binding affinity (*K*_d_) was 87 μM, a value ∼1500-fold higher than the value observed for the binding of diclofenac to site 1 of WT-TTR (*K*_d1_ = 60 nM) but only 100-fold higher than that observed for site 2 (*K*_d2_ = 1.2 μM) (49). Interestingly, the binding of diclofenac to A39D-TTR is clearly an entropy-driven process, which differs from the enthalpy-driven binding of several TTR stabilizers, which bind into the thyroxine-binding channels (49).

Furthermore, to see whether this aggregation inhibition was particular for diclofenac, we have performed aggregation kinetics in the presence of diflunisal, another TTR inhibitor (49). As seen in Fig. S6, diflunisal also inhibited aggregation of A39D-TTR. These results suggest that diclofenac and other small molecules might bind to other sites in the dimeric structure of A39D-TTR preventing its dissociation into the amyloidogenic monomers.

Taken together, these results show that the A39D-TTR forms typical amyloid fibrils, even at pH values where the WT-TTR protein does not aggregate. Furthermore, despite A39D-TTR being a dimer, nonsteroidal anti-inflammatory compounds were able to attenuate its aggregation.

## Discussion

In ATTR, the dissociation of tetramers into monomers is the rate-limiting step for TTR aggregation into amyloid fibrils, which deposit in specific tissues and organs causing their failure and the death of the patients. Microscopically, this process might involve the conversion of tetramers into dimers and dimers into monomers, which partially unfold before serving as raw material for fibril formation. Thus, tetramer stabilization through ligand binding has been one of the strategies adopted to avoid or slow down TTR amyloid fibril formation (6, 39, 50, 51). The fact that TTR has binding sites for thyroxine, its natural ligand, has contributed to the success of this strategy. Several compounds with high affinity for these sites have been designed and tested and, as they stabilize the tetramer, they hinder tetramer dissociation and aggregation. The result of these efforts led to the development of Tafamidis (2-(3,5-dichloro-phenyl)-benzox-azole-6-carboxylic acid) (51), a compound that has been used with great success in several countries, including Brazil (52). Despite its effectiveness, the treatment is still inaccessible for many patients due to its high cost, which makes other strategies necessary, such as the repositioning of drugs already in use for other purposes. In this context, there are the nonsteroidal anti-inflammatory compounds (diclofenac among them), which bind into thyroxine-binding channels (12, 13, 49), but have unwanted side effects, when used for a prolonged time (39, 53, 54) or other molecules such as Tolcapone and derivatives now being tested as ATTR drugs (55).

As mentioned, TTR dissociation into dimers and further into monomers is the trigger to TTR aggregation (25). However, the dimeric species seems to be transient, and it has not been trapped for its better characterization. To circumvent this limitation, engineered dimers of TTR have been constructed by Kelly’s group (4) and their study has shown that the dimeric AB/CD interface is weaker than the AC/BD interface, pointing towards a mechanism of TTR dissociation through the C2 crystallographic axis (AB + CD).

In the present work, we characterize structural properties of A39D-TTR, a dimeric variant of TTR found in a Brazilian family of German origin. The location and nature of the mutation explain the profound alterations in TTR quaternary structure, and this new variant is a dimer. Regarding the location of the mutation, position 39 is in the AB-loop, a region that faces the thyroxine-binding pockets and is very important to the dimer–dimer interface and formation of the tetramer, since the two dimers interact mainly *via* hydrophobic contacts mediated by AB and GH loops (56, 57). Regarding the nature of the substitution, in this new variant, there is the replacement of a small, neutral residue (alanine) by a larger, negatively-charged residue (aspartic acid), which might repel each other when in proximity.

After the identification of this new variant in our referral center at the University Hospital and due to aggressiveness of the cardiac symptoms presented by the patient, we used bioinformatics to investigate what would be the impact of this substitution in the structure of TTR as well as on its thermodynamic stability (37). The algorithm used (FoldX) showed that A39D-TTR would be a very unstable tetramer (by ∼ 11 kcal/mol) and thus one of the most unstable variants of TTR. Besides, when we modeled the substitution onto the WT structure, it was clear that there are electrostatic constraints inside the thyroxine channels due to the negative charges of the aspartic acids. Surprisingly, once purified, this new variant presented as a dimer, as evidenced by SEC (Fig. 1*A*), DLS (Fig. 1*B*), VBO binding (Fig. 1*C*), and electrophoresis (Figs. 1*D* and S2). Thus, A39D-TTR is the second dimeric variant of TTR, besides S132I-TTR (24).

It is known that crystallographic structures of several variants of TTR present subtle differences in relation to the WT structure (57, 58). The crystal structure of A39D-TTR superimposes very well with WT-TTR (Fig. 2*A*), but the position of key residues in the AB interface, namely, S135, S137, and T139, were repositioned assuming different rotamers, the same happening with R41, which moves toward D39 (Fig. 2, *D*–*F*). The first three residues are located on the DAGH β-sheet, more specifically at the H strand, a region responsible for maintaining the contacts between two monomers and thus to form the TTR dimer (Fig. 2, *E* and *F*). This region has been proposed as one of the great importance through molecular dynamic studies to amyloidogenic propensity of TTR when destabilized (59, 60).

Urea- and HHP-induced dissociation-denaturation experiments with A39D-TTR revealed the lower thermodynamic stability of the new variant (Figs. 3 and 5), as previously predicted by FoldX. Regarding HHP studies, the variant presented a p_1/2_ value of 600 bar, while p_1/2_ was equal to 1700 bar for WT-TTR (Fig. 5, *A* and *B*), consistent with its lower stability. The p_1/2_ values for the highly amyloidogenic TTR mutations A45T-TTR (formerly A25T-TTR), V50M-TTR, and L75P-TTR (formerly L55P-TTR) were, respectively, 600, 980, and 690 bar, values closely related to the one displayed by A39D-TTR (32, 34).

From the HHP data, it is possible to calculate the thermodynamic parameters (ΔGº and ΔVº of association; Equations 3 and 4) related to the tetramer-monomer and dimer-monomer equilibria (34).

**Figure 8:**
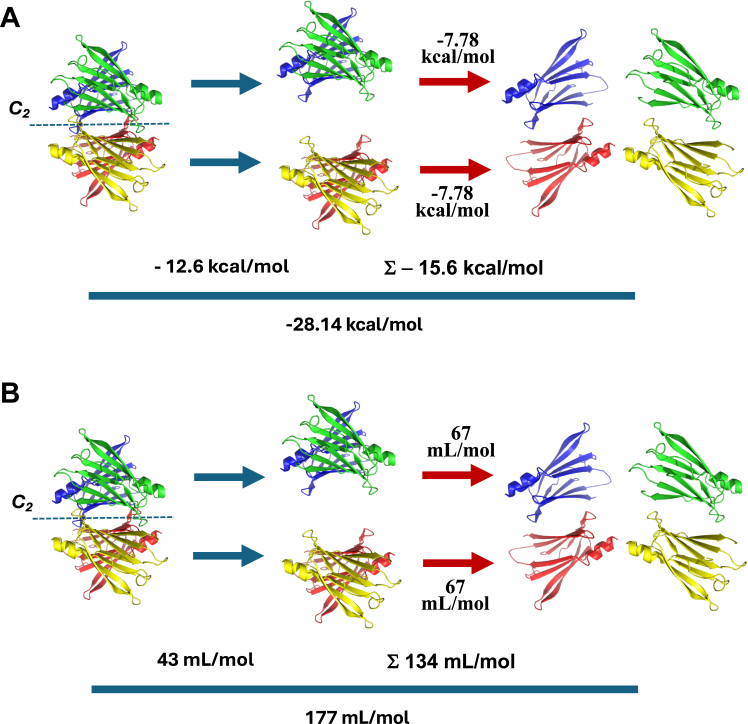
**Scheme summarizing the thermodynamic parameters related to tetramer → dimer → monomer equilibria of TTR.***A*, free energy changes for each step of TTR dissociation. The dissociation of A39D-TTR by HHP allowed us to assess the ΔG^o^ of association of −7.78 kcal/mol related to the formation of the A/B and C/D interfaces (monomer→dimer). Knowing the value for the conversion of tetramers into monomers extracted from the dissociation of the WT-TTR (−28.14 kcal/mol), we could infer the ΔG^o^ of association related to the AB/CD interface (dimer→tetramer), which equals −12.6 kcal/mol. *B*, change in volume of association for each step of TTR dissociation. The same rationale was used to assess the ΔV^o^ of association. The dissociation of the A/B and C/D interfaces is followed by a change in the volume of 67 ml/mol. The complete dissociation of tetramers into monomers furnishes a change in the volume of 177 ml/mol, which allows us to infer that the formation of the AB/CD interface is followed by a change in the volume of 43 ml/mol. The ΔGº and ΔVº values were extracted from HHP experiments according to Equations 3 and 4. Each TTR monomer is displayed in a different color.

In the case of A39D-TTR, the ΔGº of association of the two monomers forming the AB dimer is equal to −7.78 kcal/mol. Thus, we can postulate that the free energy change related to the dissociation of A/B and C/D interfaces is equal to 2 × (−7.78) kcal/mol = -15.6 kcal/mol (Fig. 8*A*). The ΔGº of association of the WT tetramer is equal to −28.14 kcal/mol. Thus, the difference in free energy change (−28.14 - (−15.6) = -12.58 kcal/mol) would be related to the formation of the *C*_*2*_ crystallographic interface, namely, the AB/CD interface, an interface which is indeed less stable than the AC/BD interface. From our experiments, we can infer that the latter interface of the tetramer is 3 kcal/mol more stable than the AB/CD interface, confirming previous data (4). Figure 8*A* summarizes all this information.

The same reasoning can be applied to the ΔVº of association (Fig. 8*B*). The volume change for the formation of A39D-TTR dimer was equal to 67 ml/mol, while the value for the formation of WT tetramers was 177 ml/mol. Thus, the formation of AB and CD dimers is accompanied by a change in the volume of 134 ml/mol (67 ml/mol × 2). Considering the whole process, the conversion of AB+CD into a tetramer is accompanied by a change in the volume of 43 ml/mol (Fig. 8*B*).

Overall, the AC/BD interface is mainly stabilized by numerous H-bonds between strands H and F from neighboring subunits. On the other hand, the AB/CD interface is maintained by hydrophobic interactions between pairs of AB and GH loops of the four subunits. Hydrophobic interactions are very sensitive to low temperatures and to HHP (41) and this explains why TTR is destabilized by the combination of these two physical agents. Indeed, HHP is more effective in denaturing TTR when applied at 1 to 4 °C (31, 32).

All these analyses reinforce the importance of the study with this dimer, which allowed us to dissect the contribution of each step in the dissociation of TTR tetramers into dimers and monomers. It must be emphasized, however, that we are extrapolating the thermodynamic parameters obtained from the A39D-TTR dimer dissociation to infer what might occur with WT-TTR dimer dissociation. The presence of the mutation might input some variations in the calculated parameters. However, knowing that position 39 is not in the monomer–monomer interface and that the crystallographic structure of the variant did not present any drastic variation, these numbers can be considered with this consideration in mind.

Regarding the urea denaturation experiments (Fig. 3), the C_m_ value obtained for A39D-TTR unfolding using circular dichroism was 2.0 M while the value extracted from changes in tryptophan emission was 2.5 M. These two values are very close, which suggests that the secondary and tertiary structures are lost almost concomitantly. However, the most interesting observation is the fact that at low concentrations of urea (up to 3 M), bis-ANS binding is enhanced, suggesting the formation of hydrophobic pockets able to accommodate this probe, a feature of molten globule structures. At these urea concentrations, A39D-TTR has already lost almost 40 to 50% of its secondary and tertiary contacts but remains as a dimer, as shown by SEC (Fig. 4*D*) and DLS. At present, we do not know which region of the proteins is lost in the presence of subdenaturing concentrations of urea and whether this altered or molten-globule dimer is aggregation prone, being an on-pathway species in the aggregation process of TTR.

As the patient with A39D-TTR presented this mutation in heterozygosis (37), we tried to recapitulate this scenario by allowing hetero-tetramers (h-T) or hetero-dimers (h-D) to form in a bacterial cell co-expressing A39D-TTR and WT-TTR proteins. This approach was used previously to investigate the formation of h-T composed of V50M and T139M monomers (61). Surprisingly, we did not observe the formation of any hetero-species, only homo-dimers, and homo-tetramers composed of A39D-TTR and WT subunits, respectively. These data were unexpected and further experiments are necessary to understand why the incorporation of a single A39D-TTR subunit renders tetramers inviable or why the incorporation of a single WT into the dimer was not noticed. Kinetics experiments could show whether differences in folding kinetics would impede the mixture of subunits. Also, using TTR-transfected mammalian cells would be the best model to determine whether hetero species composed of WT and A39D subunits would be possible.

Finally, we investigated the aggregation profile of the new variant in the absence and presence of diclofenac and diflunisal, nonsteroidal anti-inflammatory drugs that bind into the thyroxine-binding channels, stabilizing the tetramer and inhibiting TTR aggregation (12, 13). As seen in Figure 7, A39D-TTR aggregates faster and in a broader pH range when compared to the WT-TTR forming ThT-positive fibrils with the typical amyloid morphology (Fig. 7, *D* and *F*). Interestingly, increasing concentrations of diclofenac inhibited progressively the aggregation of A39D-TTR at pH 4.4 (Fig. 7*H*). Since diclofenac does not induce tetramerization of A39D (Fig. S4), which would result in thyroxine-binding sites formation, we envision that other diclofenac-binding sites might exist in the dimer. Since the A39D dimers bind bis-ANS despite the absence of the hormone channels, it is possible that other pockets indeed exist in the dimer that could accommodate these and other molecules. Further structural studies are necessary to map precisely where these sites are in the native structure of A39D-TTR, an useful information for future rational drug design against ATTR. There are other reports in the literature showing that diclofenac inhibits the aggregation of Aβ (62), α-synuclein (63), and the islet amyloid polypeptide (64), peptides that do not have binding pockets to accommodate this compound.

A39D-TTR is the second dimer of TTR. The other one is S132I-TTR, which causes FAP and FAC (65). Nonetheless, S132I- TTR, when denatured by urea, presented a C_m_ of 3.7 M monitored by tryptophan fluorescence emission, a value much higher than the one displayed by A39D-TTR and more closely related to that of the WT-TTR. S132I-TTR also presents a faster aggregation kinetics spanning a broader pH range when compared to WT-TTR as A39D-TTR does. However, differently from the A39D-TTR, aggregation of S132I-TTR was not inhibited by thyroxine or flufenamic acid, another nonsteroidal anti-inflammatory drug (24). These differences in behavior show how heterogenous can be these two dimers and the impact of different mutations on the overall structure of TTR. Position 132 is in GH loop, while position 39 is in AB loop. These two loops are very important to dimer–dimer interaction to form the tetramer (56, 57). Thus, mutations in these loops are expected to impact TTR stability considerably and they do impede the dimers to tetramerize.

The importance of TTR loops have been addressed since they can impact the global TTR structure and stability (66, 67, 68, 69, 70). The mutation D38G was described in a patient with ocular leptomeningeal amyloidosis. Position 38 is very close to ours and is also located in AB loop (23). This substitution was so drastic that D38G-TTR is a monomer in solution. D38 makes several important electrostatic contacts at the dimer–dimer interface (57), and the replacement by glycine breaks this array of electrostatic interactions necessary for tetramer formation.

## Conclusion

The present study dissected the thermodynamic stability and aggregation properties of a novel dimeric and highly amyloidogenic variant of TTR, namely, A39D-TTR. HHP-induced denaturation studies allowed the calculation of ΔG^o^ and ΔV^o^ of association for each step in TTR tetramer→ dimer→ monomer dissociation. Urea-induced unfolding allowed the description of a molten globule dimer, which binds high amounts of bis-ANS. The role of this altered dimeric species in the TTR aggregation process needs further investigation. The aggregation process of A39D-TTR was inhibited by diclofenac and diflunisal in a concentration-dependent manner, which suggests the existence of additional binding sites for compounds in TTR structure besides the thyroxine-binding channels, thereby opening new avenues for the description of new compounds for the treatment of ATTR.

## Experimental procedures

### Expression and purification of TTR in *E. coli*

Both expression and purification of TTR were carried out following the methodology previously established in the work of Lashuel (71). Protein expression was performed by transforming competent *Escherichia coli* BL21 DE3 with the A39D-TTR and WT-TTR plasmid in medium with the antibiotic ampicillin (100 μg/ml) to select for the expression of WT-TTR and kanamycin (100 μg/ml) to select for A39D-TTR expression.

TTR concentration was determined spectrophotometrically at 280 nm using the molar extinction coefficient of 77,600 mol^−1^ cm^−1^ for WT-TTR and 38,800 mol^−1^ .cm^−1^ for A39D-TTR.

### Coexpression of A39D-TTR and WT-TTR in bacterial cells

To investigate the formation of hetero-tetramers and hetero-dimers, competent *E. coli* BL21 DE3 cells were transformed with both plasmids, which bear different resistance to antibiotics as stated above. Bacterial cells resistant to both antibiotics were grown for further TTR purification (see scheme in Fig. 6*A*) using the same protocol already established (71). In the last step of purification (SEC), the peaks corresponding to the dimer and tetramer were carefully collected and sent to mass spectrometry for protein identity assignment. This experiment was repeated three times with the same results.

### SEC to investigate the oligomerization state of A39D-TTR in low urea concentrations and in the presence of diclofenac

The 120 ml HiLoad 16/60 Superdex 75 prep grade column was connected to an ÄKTA start system (GE Healthcare Life Sciences) for SEC sample purification runs for use in the other experiments, as well as in the experiments related to the coexpression protocol, after the column was equilibrated with a PBS solution (pH 7.4).

A 25 ml Superdex 75 10/300 Gl prep grade column (Thermo Fisher Scientific Inc.) connected to a Shimadzu system for HPLC runs was equilibrated with 25 mM Tris HCl, 100 mM KCl, and 1 mM EDTA (pH 8.0) as running buffer to evaluate both purity and oligomerization profile of the WT-TTR and A39D-TTR samples post purification in the 120 ml HiLoad 16/60 Superdex 75 prep grade column. In addition, 25 mM Tris HCl, 100 mM KCl, and 1 mM EDTA (pH 8.0) buffer with either 20 μM diclofenac or 2.5 M urea was used when indicated. The following proteins were used as molecular weight standards: α-globulin (158 kDa), ovalbumin (44 kDa), and myoglobin (17 kDa).

### Crystallization, data collection, and structure refinement of A39D-TTR

The mutant A39D-TTR was crystallized by hanging-drop vapor diffusion. Crystals were grown for 1 week at 18 °C in 100 mM HEPES (pH 7.5), 200 mM CaCl_2_, and 28% PEG400 using 10 mg/ml A39D-TTR. The crystal was cooled, and X-ray diffraction data were collected at beamline I04-1 of Diamond Light Source (λ = 0.9200 Å). The diffraction data were processed automatically using Xia2 (72) up to 1.37 Å resolution. The structure of A39D-TTR was determined by molecular replacement with Phaser using the apo form of human TTR as a search model (Protein Data Bank entry 3CFM). The refinement was performed using Pheni and Coot. The electron density for the A39D-TTR mutation was identifiable in the first map. Validation of the structural model was performed with Molprobity.

### Dynamic light scattering

DLS was performed using ZetaPALS equipment (Brookhaven Instruments Corp.). For particle-size estimation in native conditions, A39D-TTR and WT-TTR samples were diluted in 25 mM Tris HCl, 100 mM KCl, and 1 mM EDTA (pH 8.0), to 10 μM protein concentration. In experiments made to estimate particle size with and without 1 M and 2.5 M urea concentrations, A39D-TTR and WT-TTR at 10 μM protein concentration were diluted in 25 mM Tris HCl, 100 mM KCl, and 1 mM EDTA (pH 8.0) buffer and the desired urea concentration. Analyses were performed at room temperature using an Eppendorf UVette cuvette with a 10 mm optical path. The results were obtained from an average of 5 runs, being represented by the average of the measurements.

### Circular dichroism spectropolarimetry

For protein secondary structure determinations, far-UV (190–260 nm) spectra of the samples were recorded at 25 °C using a Hirascan CD spectrometer. Spectra were recorded in a 1 mm quartz cell with 0.5 nm intervals and a speed of 70 nm/min. Each spectrum represents the average of three scans.

For urea-induced denaturation experiments, the concentrations of WT-TTR and A39D-TTR were, respectively, 2 and 4 μM in increasing concentrations of urea (0–8 M) diluted in PBS buffer pH 7.4. The data were analyzed by first subtracting the spectrum of the buffer with the same concentration of urea from the spectrum containing the proteins at the same concentration of denaturant.

To estimate the content of the secondary structure of the proteins in each concentration of urea, the degree of denaturation (α) was calculated according to Equation 1 using the signal at 215 nm.

(1) α=[(υm−υi)][(υf−υi)]

In this equation, υi and υf are the signals at 215 nm at 0 and 8 M urea, respectively, while υm is the signal in the presence of different concentration of urea. Since denaturation of WT-TTR is incomplete even in the presence of 8M urea, when necessary, we used the signal of A39D-TTR at 215 nm in the presence of 8M urea to estimate its partial degree of unfolding.

### Tryptophan fluorescence emission

TTR solutions were incubated for 96 h at 25 °C in increasing concentrations of urea (0–8 M). After this time, tryptophan fluorescence emission was measured by exciting the samples at 280 nm and collecting emission from 300 to 400 nm in an ISSPC1 spectrofluorimeter (ISS Inc.) at room temperature. The spectrum of the buffer in the presence of each urea concentration was always subtracted from its corresponding spectrum in the presence of protein.

To evaluate the extent of unfolding in the presence of urea, the center of mass of tryptophan emission (C_m_) was calculated according to Equation 2, where Fi is the fluorescence emission for a certain wavelength υi and the summation is carried out over the measurement interval of the fluorescence values. The changes in C_m_ were converted into degree of denaturation according to Equation 1, assuming that υi and υf are, respectively, the C_m_ in 0 M and 8 M urea, while υm is the value at any urea concentration. Again, when necessary, the C_m_ of the completely unfolded A39D-TTR was used to estimate the partial unfolding of the WT-TTR. The same equations were used for measuring changes in tryptophan fluorescence emission induced by HHP.

(2) $$C_{m}=\sum \left(\upsilon i.Fi\right)/\sum Fi$$

The experiments were repeated three times and the results shown are the means of the three measurements. The protein concentrations were 1 μM WT-TTR and 1 and 10 μM A39D-TTR.

### Bis-ANS and VBO fluorescence emission

Bis-ANS (4,4′-dianilino-1,1′-binaphthyl-5,5′-disulphonic acid, dipotassium salt) and VBO (2-[(3,5-dichlorophenyl)amino]benzoic acid) emission spectra were recorded by exciting the sample at 385 nm and 320 nm and collecting the emission from 435 nm to 580 nm and from 450 to 600 nm, respectively, in an ISSPC1 spectrofluorimeter (ISS Inc.) (40). The proteins were used at a final concentration of 2 μM WT-TTR and 4 μM A39D-TTR and the probes were both used at a final concentration of 10 μM for the analyses presented in Figure 1*C* (VBO fluorescence) and Figure 4 (bis-ANS fluorescence). The kinetic of A39D and WT-TTR binding to bis-ANS at 2.5 M urea concentration was measured exciting the samples at 385 nm and monitoring the fluorescence emission at 485 nm for a total time of 60 min.

To evaluate whether VBO could induce the tetramerization of A39D-TTR at higher protein concentrations, we preincubated A39D-TTR using a range of 2.5 to 40 μM protein with either 1:1 or 1:2 proportion of ptn:VBO diluted in 25 mM Tris HCl, 100 mM KCl, and 1 mM EDTA (pH 8.0) for 2 h prior to fluorescence measurements. We used WT-TTR (10 μM) with VBO (20 μM and 40 μM) as a control measurement.

The experiments were repeated three independent times and the results shown are the means of the three measurements.

### HHP-induced unfolding

HHP experiments were conducted in 50 mM Tris, 100 mM KCl buffer at pH 7.0. The pressure was increased in steps of 200 bar. Tryptophan fluorescence emission was collected between 300 and 400 nm, with excitation at 280 nm in an ISSPC1 spectrofluorimeter (ISS Inc., Champaign). The experiments were carried out at 1 °C to favor TTR dissociation and unfolding by HHP. The protein concentrations used were 1 μM and 10 μM for A39D-TTR and 1 μM for WT-TTR.

To calculate the thermodynamic parameters of association, the curves with the degree of denaturation (α) *versus* pressure were converted to log scale according to Equations 3 and 4, for the dissociation of WT-TTR (tetramer to monomers) and A39D-TTR (dimer to monomers), respectively. The standard volume change of association (ΔVº) and the free energy change of association (ΔGº) are, respectively, the slope and intercept on the y axis (34, 73). In these equations, C is the protein concentration used in the experiment, T the temperature (in K), and R (cal.K^-1^.mol^-1^) the gas constant.

(3) $$\ln\left[\frac{{\left(\alpha_{p}\right)}^{4}}{1-\;\alpha_{p}}\right]=p\left(\frac{\Delta V}{RT}\right)\;+\;\ln\left(\frac{K_{d}}{256C^{3}}\right)$$

(4) $$\ln\left[\frac{{\left(\alpha_{p}\right)}^{2}}{1-\;\alpha_{p}}\right]=p\left(\frac{\Delta V}{RT}\right)\;+\;\ln\left(\frac{K_{d}}{4C}\right)$$

### Aggregation assays

In order to analyze the aggregation pH range of A39D-TTR and WT-TTR, 10 and 5 μM protein, respectively, were diluted in 100 mM sodium acetate, 100 mM KCl, 5 mM sodium azide to pH values 4.0 to 5.2; 100 mM MES, 100 mM KCl, 5 mM sodium azide, for pH 5.4 to 6.4, and 100 mM Tris HCl, 100 mM KCl, 5 mM sodium azide, for pH values 7.0 and 7.4.

To analyze the influence of a protein stabilizer on the A39D-TTR aggregation, diclofenac and diflunisal were dissolved in a DMSO in stock solutions of 5, 10, and 15 mM. WT- and A39D-TTR were 10 and 20 μM, respectively, and incubated for 2 h with increasing concentrations of diclofenac in a solution of 10 mM Tris HCl, 100 mM KCl, and 5 mM sodium azide pH 8.0 (49). Then, these samples were diluted 1:1 with acidification buffer (200 mM sodium acetate, 100 mM KCl, and 5 mM sodium azide pH 4.4) to trigger the kinetics.

Aggregation kinetics were performed at 37 °C for 24 h, 48 h, and 72 h without agitation in a microcentrifuge tube. Turbidity at 330 nm was measured in a spectrophotometer (SpectraMAx Plus 384). The solutions were shaken gently before measurements. Short kinetics (up to 4h) were performed in a 96-well plate on a Shimadzu UVmini-1240 spectrophotometer. These experiments were repeated three independent times and the results shown are the average and SD of the three measurements.

### ThT binding assays

ThT (Sigma, product no. T3516) was dissolved in ultrapure water and filtered through a 0.2 μm syringe filter. Then, the concentration was determined using an extinction coefficient of 36 mM^−1^ .cm^−1^ at 412 nm. As the affinity of ThT to amyloid fibrils generally decreases at acidic pH (74), after incubating the protein samples at acidic pH values for the indicated times at 37 °C, samples were vortexed to achieve a homogeneous solution. Aliquots of the samples with a final concentration of 1 μM protein were diluted in 25 μM ThT in 200 mM Tris HCl, 100 mM KCl (pH 8.0) in a final volume of 300 μl (70).

Samples were excited at 450 nm and emission collected at 482 nm in a plate reader (SpectraMax Gemini EM). These experiments were repeated three times and the result shown denotes the average of the three measurements, as well as the SDs.

### Transmission electron microscopy

Aggregated suspensions were absorbed onto 200-mesh carbon-coated copper grids for 5 min and then blotted to remove excess material. Negative staining was performed by adding 5 μl of 1% (w/v) uranyl acetate. Samples were dried in air for 3 min. The grids were imaged with a ZEISS EM 900 (Carl Zeiss Inc.) electron microscope. The protein concentration was 5 μM for WT-TTR and 10 μM for A39D-TTR.

### Mass spectrometry assays

Samples were diluted to 1 μM in a solution of 3% acetonitrile and 0.1% formic acid, and 5 μl were injected into the Waters Nano Acquity system (Waters), which consists of a nanochromatographic system with pre -Waters Symmetry C18 column (180 μm X 20 mm, 5 μm) and an HSS T3 C4 analytical column (75 μm X 100 mm, 1.7 μm Waters). The sample was injected and desalted in the pre-column and then eluted from the analytical column at a flow rate of 0.5 μl/min, in a step of 30% acetonitrile containing 0.1% formic acid.

Mass spectra were acquired on a Synapt HDMS mass spectrometer (Waters) in positive mode, and electrospray ionization was performed using 4000 V, source temperature 80 °C and cone voltage 40 V.

Data acquisition and instrument control were conducted in the MassLynx program (Version 4.1, Waters). Chromatographic runs were performed with a range of 50 to 2000 mass/charge ratio (m/z), using 1 s scan intervals applied throughout the chromatographic process.

The average mass value of each protein was determined manually using the MassLynx program (Version 4.1, Waters) by deconvolution of charge states with a maximum entropy algorithm (MaxEnt 1, Waters). Spectra were calibrated in real-time acquisition with the LockSpray system (Waters) every 30 s.

### Isothermal titration calorimetry

The dissociation constant (*K*_d_) for diclofenac binding to A39D-TTR was assessed in a MicroCal ITC200. A solution of diclofenac (500 μM in 10 mM Tris HCl, 100 mM KCl, 0.5% DMSO, pH = 8.0) was prepared and titrated into an ITC cell containing A38D-TTR (10 μM in 10 mM Tris HCl, 100 mM KCl, 0.5% DMSO, pH = 8.0). The initial injection of 0.4 μl was followed by 19 injections of 2 μl each at 25 °C, and the interval between injections was 180 s with agitation of 750 rpm and a reference power of 8 μcal/sec. The reference cell was filled with ultrapure water. Integration of the thermogram after subtraction of blanks yielded a binding isotherm that were better fitted to a model of one binding site with the Origin 7.0 program (MicroCal). The *K*_d_ value was calculated based on the K_a_ value.

### Statistics

Statistical analysis was performed using GraphPad Prism Software (https://www.graphpad.com/), version 8.0 (GraphPad). For statistical analyses, *p* values ;< 0.05 were considered significant. For the aggregation kinetics experiments by absorbance and ThT with varying diclofenac concentrations or different pH values, one-way ANOVA analysis with multiple comparisons and Tukey's correction was used. For aggregation kinetics experiments comparing A39D-TTR and WT-TTR only, we used a Student's *t* test for paired samples.

## Data availability

All data supporting the results of this study are available in the article when requested.

## Supporting information

This article contains supporting information.

## Conflict of interest

The authors declare that they have no conflicts of interest with the contents of this article.
