# Supplementary material for: Structural and thermodynamic characterization of a highly amyloidogenic dimer of transthyretin involved in a severe cardiomyopathy
Source: J Biol Chem. 2024 Jun 24;300(8):107495. doi: 10.1016/j.jbc.2024.107495 (PMC11293521; doi:10.1016/j.jbc.2024.107495)
Supplement: Supplemental Figures [file mmc1.docx]

***Supplementary Figure 1:*** **DLS correlation curves for A39D-TTR (A) and WT-TTR (B) for the data presented in Figure 1 B*.***

***Supplementary Figure 2:***  **SDS-PAGE (18%) for WT-TTR and A39D-TTR at increasing protein concentrations as displayed above each lane of the gels.** At 40 μM, the % of each protein species in WT- and A39D-TTR are: WT-TTR: 2.95% of tetramers, 2.43% of dimers and 94.62% of monomers; A39D-TTR: 0.62% of tetramers, 2.84% of dimers and 96.54% of monomers.

***Supplementary Figure 3:*** **Measuring VBO binding to WT-TTR and A39D-TTR at increasing protein concentrations as stated in the Figure**. Note that VBO does not bind to A39D-TTR even at high protein concentrations suggesting that the variant does not form a tetramer under this condition. [VBO] was kept either 1:1 or 1:2 (ptn:VBO) as displayed in the Figure.

***Supplementary Figure 4:*** **Diclofenac does not induce the tetramerization of A39D-TTR.** A and B (magnification of the 18 – 23 min area from panel A) depict the elution profiles by SEC performed on an SD-75 column equilibrated with 25 mM Tris HCl, 100 mM KCl and 20 µM diclofenac (pH 8.0) at a constant flow of 0.5 mL/min. A39D-TTR, blue line; WT-TTR, black line. Both proteins were previously incubated with 20 µM diclofenac for 2 h before injection into the column.

***Supplementary Figure 5:*** Assessing the dissociation constant of diclofenac to **A39D-TTR by ITC.** A solution of diclofenac (500 μM) was titrated into an ITC cell containing A39D-TTR (10 μM) with a total of 20 injections of 2 μL each at 25 ºC.

***Supplementary Figure 6:*** **A39D-TTR aggregation is inhibited by diflunisal.** Aggregation kinetics with WT- (**A;** 5 µM) and A39D-TTR (**B**; 10 µM) at pH 4.4, for 72 h in the absence (blue bars) or in the presence of increasing concentrations of diflunisal (5 µM diflunisal, red; 10 µM diflunisal, green; 20 µM diflunisal, purple and 50 µM diflunisal, orange). Turbidity at 330 nm and ThT binding data were normalized considering as 1 the highest aggregation values obtained either with WT- or A39D-TTR in the absence of diflunisal. Data with p values < 0.05 were considered significant. Asterisks: * = p < 0.05; ** = p < 0.01; *** = p < 0.001 **** = p < 0.0001.
