# Supplementary material for: Structural and thermodynamic characterization of a highly amyloidogenic dimer of transthyretin involved in a severe cardiomyopathy
Source: J Biol Chem. 2024 Jun 24;300(8):107495. doi: 10.1016/j.jbc.2024.107495 (PMC11293521; doi:10.1016/j.jbc.2024.107495)

Supplementary Figures

Supplementary Figure 1

A A39D-TTR

B WT-TTR

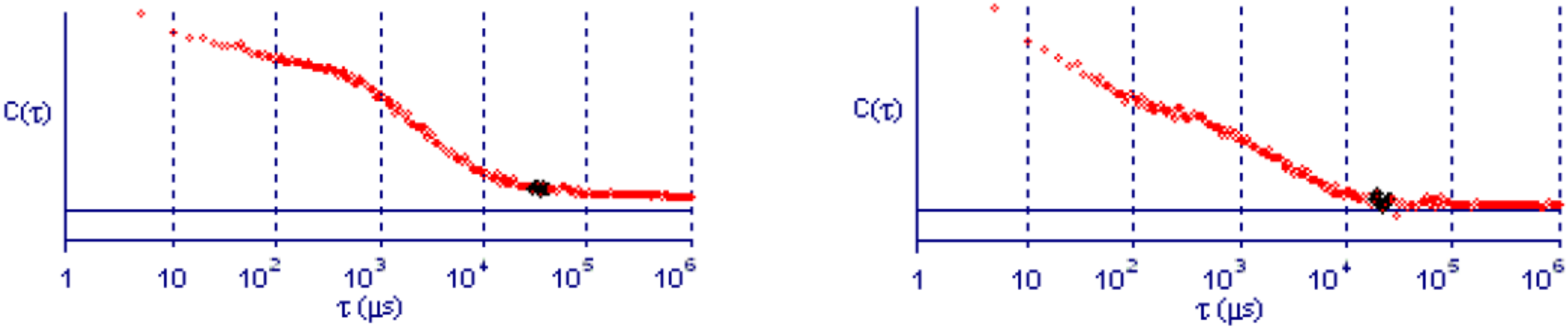

Supplementary Figure 2

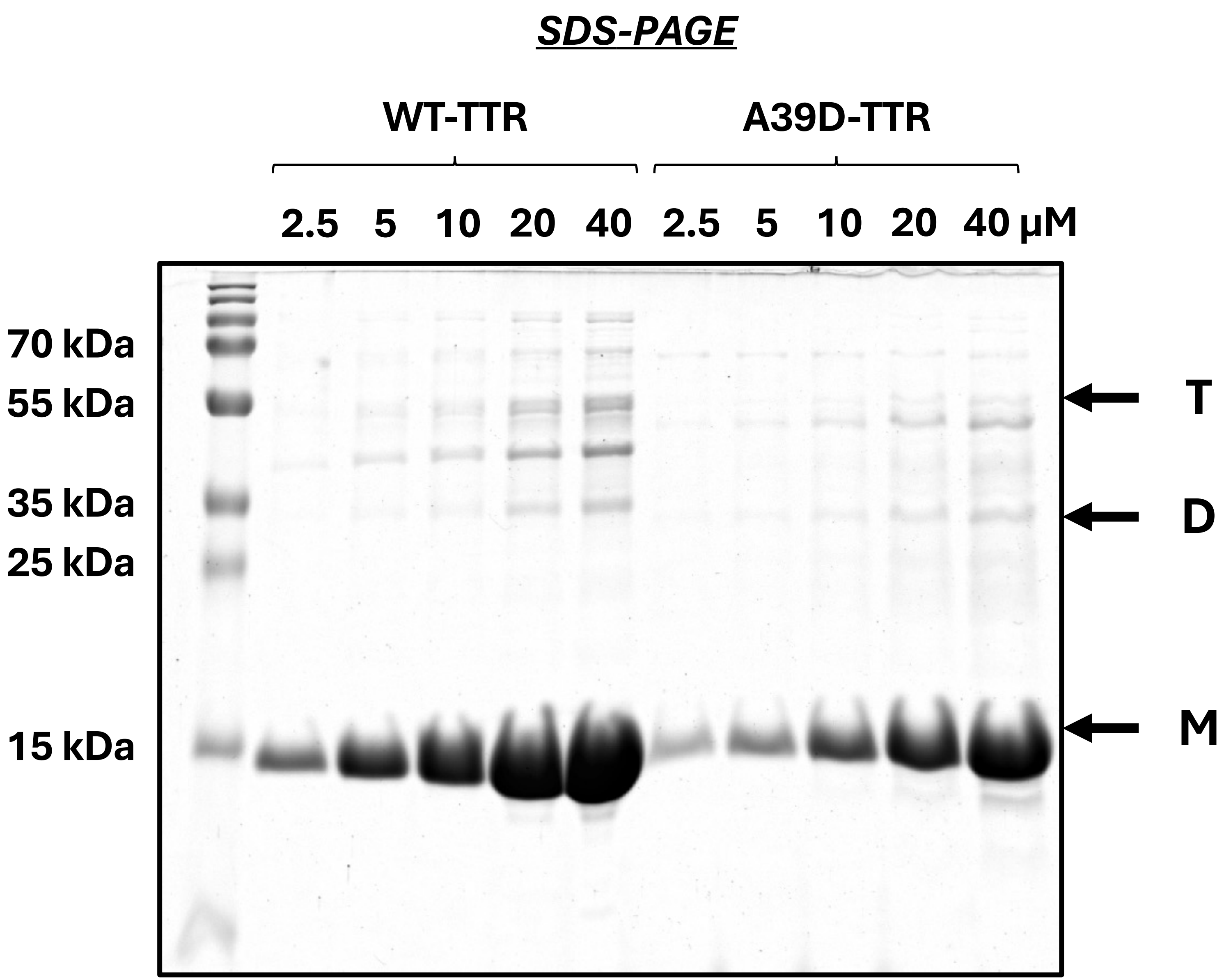

Supplementary Figure 3

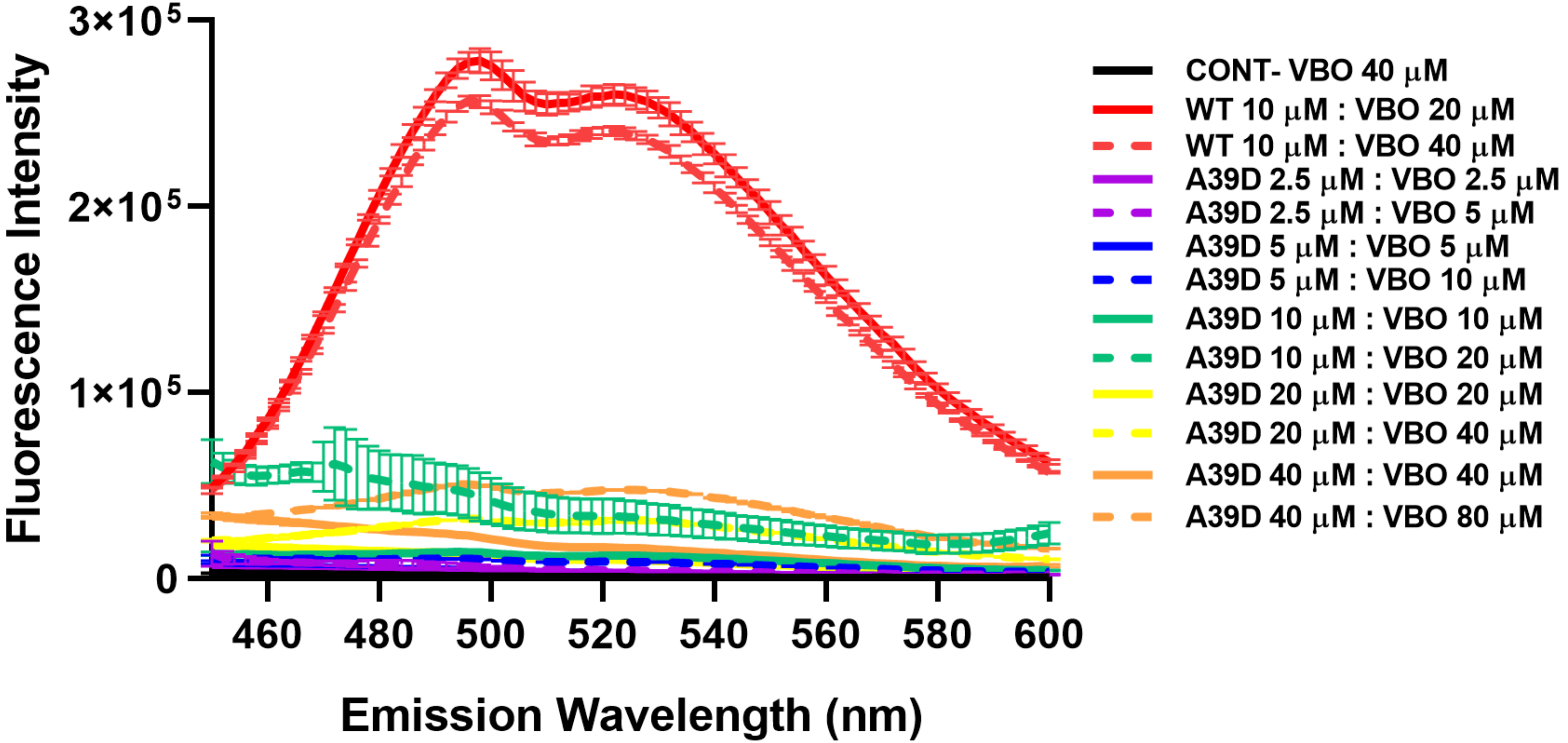

Supplementary Figure 4

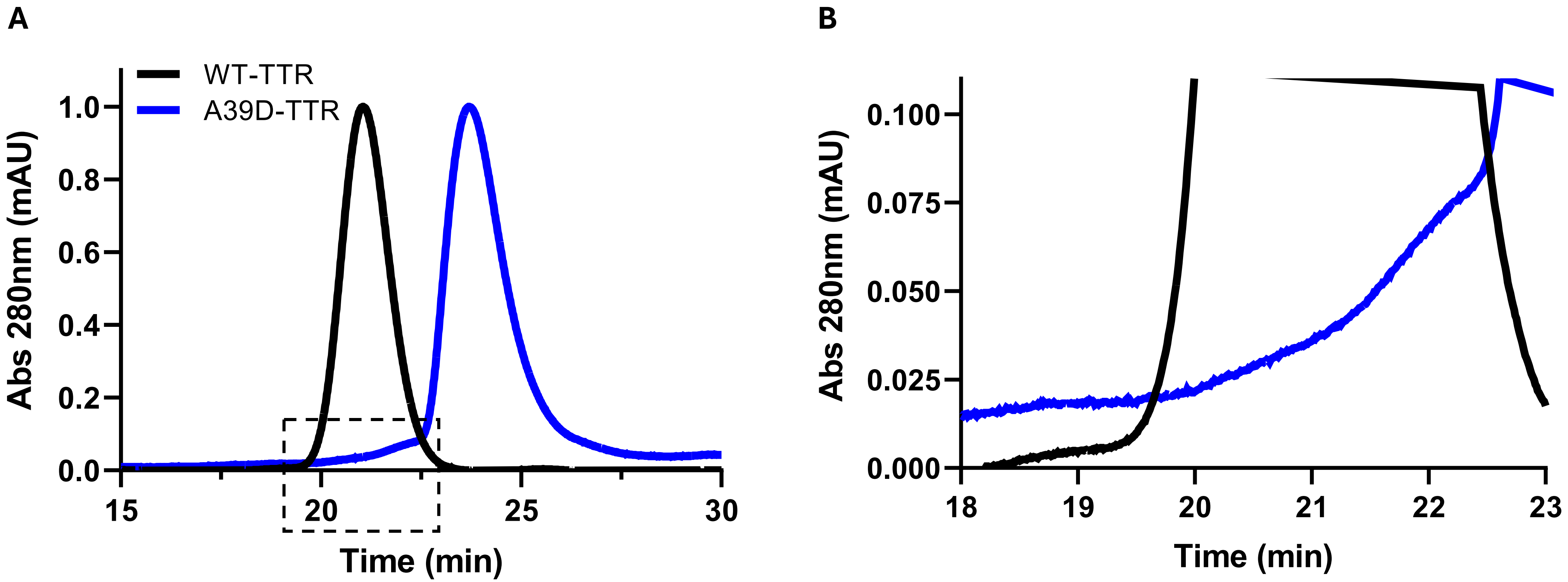

Supplementary Figure 5

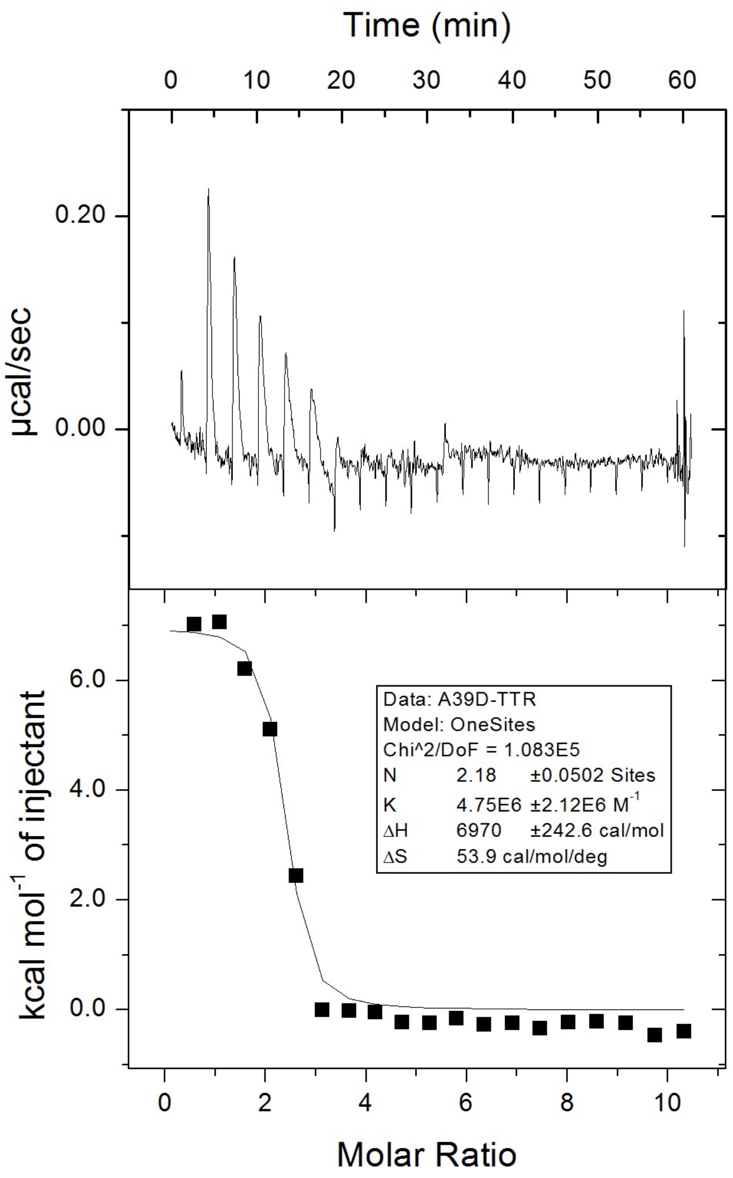

Supplementary Figure 6

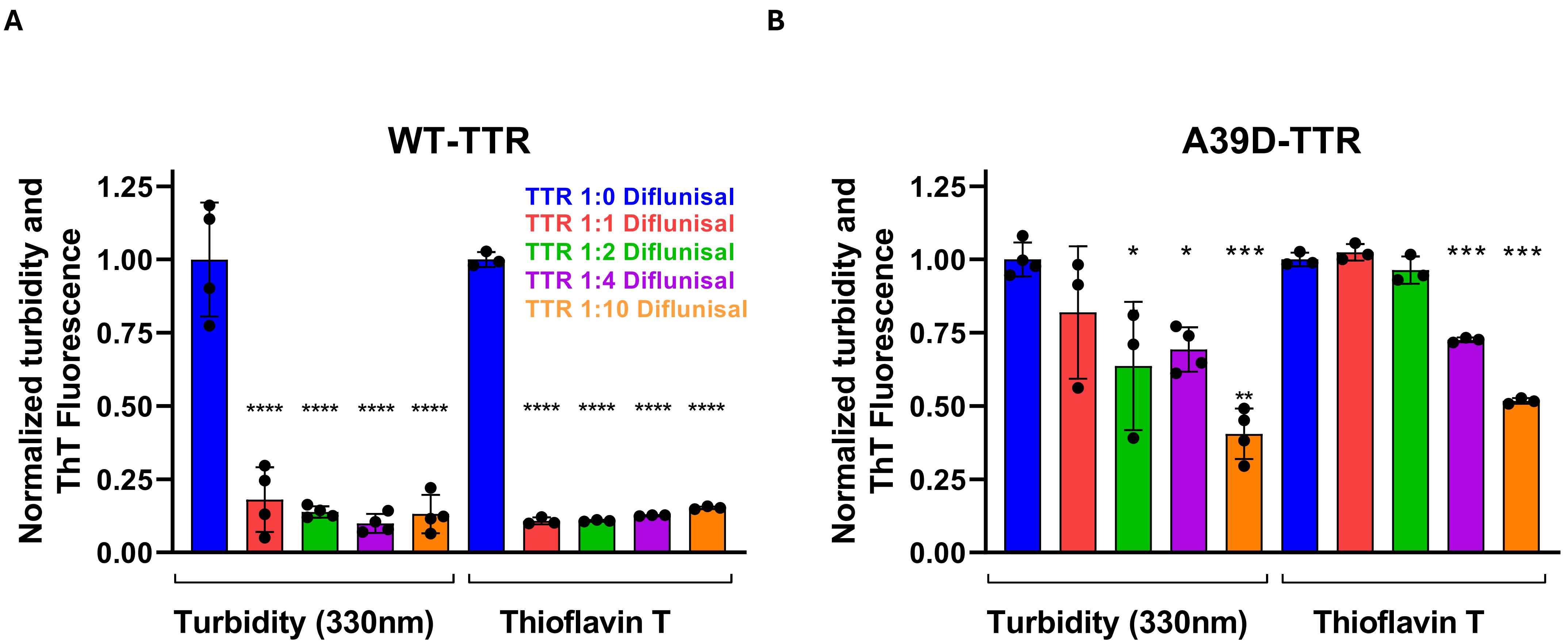

Supplement: Supplemental data [file mmc2.pdf]
